# *DETORQUEO*, *QUIRKY*, and *ZERZAUST* Represent Novel Components Involved in Organ Development Mediated by the Receptor-Like Kinase STRUBBELIG in *Arabidopsis thaliana*

**DOI:** 10.1371/journal.pgen.1000355

**Published:** 2009-01-30

**Authors:** Lynette Fulton, Martine Batoux, Prasad Vaddepalli, Ram Kishor Yadav, Wolfgang Busch, Stig U. Andersen, Sangho Jeong, Jan U. Lohmann, Kay Schneitz

**Affiliations:** 1 Entwicklungsbiologie der Pflanzen, Wissenschaftszentrum Weihenstephan, Technische Universität München, Freising, Germany; 2 Max Planck Institute for Developmental Biology, Department of Molecular Biology, AG Lohmann, Tübingen, Germany; 3 Section of Cell and Developmental Biology, University of California San Diego, La Jolla, California, United States of America; 4 Center for Organismal Studies, University of Heidelberg, Heidelberg, Germany; The Salk Institute for Biological Studies, United States of America

## Abstract

Intercellular signaling plays an important role in controlling cellular behavior in apical meristems and developing organs in plants. One prominent example in *Arabidopsis* is the regulation of floral organ shape, ovule integument morphogenesis, the cell division plane, and root hair patterning by the leucine-rich repeat receptor-like kinase STRUBBELIG (SUB). Interestingly, kinase activity of SUB is not essential for its in vivo function, indicating that SUB may be an atypical or inactive receptor-like kinase. Since little is known about signaling by atypical receptor-like kinases, we used forward genetics to identify genes that potentially function in *SUB*-dependent processes and found recessive mutations in three genes that result in a *sub*-like phenotype. Plants with a defect in *DETORQEO* (*DOQ*), *QUIRKY* (*QKY*), and *ZERZAUST* (*ZET*) show corresponding defects in outer integument development, floral organ shape, and stem twisting. The mutants also show *sub*-like cellular defects in the floral meristem and in root hair patterning. Thus, *SUB*, *DOQ*, *QKY*, and *ZET* define the *STRUBBELIG-LIKE MUTANT* (*SLM*) class of genes. Molecular cloning of *QKY* identified a putative transmembrane protein carrying four C_2_ domains, suggesting that QKY may function in membrane trafficking in a Ca^2+^-dependent fashion. Morphological analysis of single and all pair-wise double-mutant combinations indicated that *SLM* genes have overlapping, but also distinct, functions in plant organogenesis. This notion was supported by a systematic comparison of whole-genome transcript profiles during floral development, which molecularly defined common and distinct sets of affected processes in *slm* mutants. Further analysis indicated that many *SLM*-responsive genes have functions in cell wall biology, hormone signaling, and various stress responses. Taken together, our data suggest that *DOQ*, *QKY*, and *ZET* contribute to *SUB*-dependent organogenesis and shed light on the mechanisms, which are dependent on signaling through the atypical receptor-like kinase SUB.

## Author Summary

Plant organs, such as flowers or leaves, are made up of distinct cell layers. Although communication across these cell layers is essential for organ development, we have only recently gained some insight into the underlying mechanisms. Receptor-like kinases are cell-surface receptors that perceive and relay intercellular information. In *Arabidopsis*, the receptor-like kinase STRUBBELIG is required for inter–cell-layer communication during floral development, amongst other functions; little is known, however, concerning its exact signaling mechanism. Here, we identified three new genes called *DETORQUEO*, *QUIRKY*, and *ZERZAUST*. Plants defective in any of these genes strongly resemble the *strubbelig* mutant, both at the whole-organ and cellular levels. Thus, all four genes may share or contribute to a common signaling pathway essential for plant morphogenesis. Analyses revealed complex interactions between the genes, indicating that each has additional and distinct activities. We provide the molecular nature of *QUIRKY*; the encoded protein is likely membrane-localised and predicted to require Ca^2+^ for activity. In light of analogous animal models, we speculate that *QUIRKY* facilitates transport of molecules to the cell boundary and may support a *STRUBBELIG*-related extracellular signal. These results open new inroads into a molecular understanding of inter-cellular communication during flower development.

## Introduction

How intercellular communication mechanisms coordinate the activities of cells during organogenesis is an important topic in biology. In higher plants shoot apical meristems and floral meristems are the ultimate source of above-ground lateral organs, such as leaves, flowers, and floral organs [1]. Meristems are organised into three distinct meristematic or histogenic layers, called L1, L2, and L3 [2], and cells of all histogenic layers contribute to organogenesis [3],[4]. The L1 layer gives rise to the epidermis while the L2 and L3 layers contribute to internal tissues. In *Arabidopsis* ovules, for example, the integuments that eventually develop into the seed coat are entirely made up of L1-derived cells, while L2 cells generate the inner tissue [5].

Classic studies have demonstrated that meristematic layers communicate [6],[7], but it is only recently that the biological relevance and the molecular mechanisms are being elucidated [8]–[11]. For example, work on the receptor-like kinase (RLK) BRASSINOSTEROID INSENSITIVE 1 (BRI1) has provided evidence that the epidermis both promotes and restricts organ growth [12]. Furthermore, microsurgical experiments indicated that the epidermis also maintains cell division patterns in subtending layers [13]. These are but two examples that highlight the importance of the epidermis and inwards-oriented signaling in this inter-cell-layer cross-talk required for correct organ size and shape. At the same time, radial outward-oriented signaling also takes place during organogenesis. Known scenarios include transcription factors or small proteins that are synthesized in inner layers and move outwards into overlaying cell layers in a controlled fashion [14]–[17]. The so far best-characterised case of such a movement underlies radial patterning of the root [18],[19]. In addition, the epidermally-expressed RLKs CRINKLY4 (CR4) from corn or its *Arabidopsis* homolog ACR4 are necessary for epidermis development and may receive signals from underlying cell layers [20]–[24].

Inter-cellular communication during floral morphogenesis in *Arabidopsis* also depends on signaling mediated by the leucine-rich repeat transmembrane receptor-like kinase (LRR-RLK) STRUBBELIG (SUB) [25]. Analysis of *sub* mutants indicated that *SUB* is required for proper shaping of floral organs such as carpels, petals and ovules. At the cellular level *SUB* participates in the control of cell shape and/or the orientation of the cell division plane in floral meristems and ovules. In addition, *SUB*, also known as *SCRAMBLED* (*SCM*), affects specification of hair cells in the root epidermis [26],[27]. Recent evidence suggests that the SUB protein is confined to interior tissues in floral meristems, developing ovules and young roots although *SUB* mRNA is monitored throughout those organs [28]. In particular functional SUB:EGFP fusion protein is absent from cells that show a mutant phenotype in *sub* mutants, but can either be found in adjacent cells, as in floral meristems and ovules, or in cells that are separated from mutant cells by two cell diameters, like in the root. The non-cell-autonomous effects of *SUB* were corroborated by an analysis of *sub-1* plants expressing a functional *SUB:EGFP* transgene under the control of different tissue-specific promoters. Thus the data indicate that *SUB* undergoes posttranscriptional regulation, acts in a non-cell-autonomous fashion and mediates cell morphogenesis and cell fate across clonally distinct cell layers in an inside-out fashion [28]. The SUB protein is a member of the LRRV/STRUBBELIG-RECEPTOR FAMILY (SRF) family of receptor-like kinases [29],[30]. It is predicted to carry an extracellular domain with six leucine-rich repeats, a transmembrane domain, and a cytoplasmic intracellular domain with the juxtramembrane and kinase domains. Interestingly, phosphotransfer activity of the SUB kinase domain is not essential for its function *in vivo* [25] and thus SUB seems to belong to the family of atypical or “dead” receptor kinases [31],[32].

Very little is known regarding signaling through atypical receptor-like kinases in plants [31]. In addition, it remains to be understood how cellular morphogenesis is coordinated across cell layers [8]–[11]. It is therefore of great interest to investigate the molecular basis of *SUB* signaling and function. Here we present the identification and analysis of three genetic factors that may relate to *SUB* signaling. Our results show that mutations in *QUIRKY* (*QKY*), *ZERZAUST* (*ZET*), and *DETORQUEO* (*DOQ*) result in a *sub*-like phenotype. Molecular cloning of *QKY* revealed that the predicted QKY protein is likely a transmembrane protein with four C_2_ domains indicating a role for QKY in Ca^2+^-dependent signaling. Global gene expression profiling of the mutants corroborates the morphological analysis but also suggests additional and distinct roles for each gene. Furthermore, the data indicate that *SUB* signaling plays previously unknown roles in cell wall and stress biology.

## Results

### Isolation of *sub*-Like Mutants

**Figure 1:**
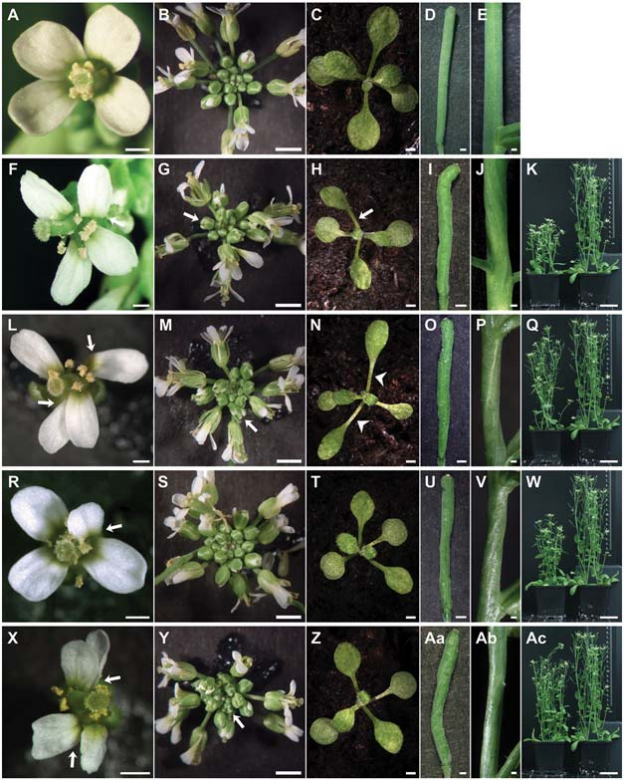
Comparison of the overall above-ground morphology of *sub-1*, *doq-1*, *qky-8*, and *zet-2* mutants. The given stages correspond across equivalent panels. (A–E) Wild-type L*er*. (F–K) *sub-1*. (L–Q) *doq-1*. (R–W) *qky-8*. (X-Ac) *zet-2*. (A, F, L, R, X) An open stage 13 flower from a 30-day old plant. Note the misorientation of petals due to twisting in the basal end of the petal structure (arrows). (F, R, X) Petals can also show small notches. (B, G, M, S, Y) Top view of a 30-day inflorescence. (G, M, S, Y) Flower phyllotaxis is irregular. Arrows mark prematurely opened flower buds. (C, H, N, T, Z) Top view of a 12-day rosette. (H, N) Leaf petioles can be twisted (arrows). (N) *doq-1* leaves have longer petioles and narrow blades (arrow heads). (D, I, O, U, Aa) Morphology of mature siliques. Typical twisting observed in (I) *sub-1*, (U) *qky-8* and (Aa) *zet-2* mutants. *doq-1* exhibits more subtle twisting in both (O) siliques and (P) stems. (E, J, P, V, Ab) A lateral view of a section of stems from a 30-day plant. (V) *qky-8* and (Ab) *zet-2* mutant show twisting in stems equivalent to (J) *sub-1* plants. (K, Q, W, Ac) Plant height of mutants (left) in comparison to Ler (right). (K) *sub-1* and (W) *qky-8* mutants show a clearly reduced plant height. (Q) The *doq-1* and (Ac) *zet-2* mutants show only a slight reduction. Scale bars: (A, D, E, F, I, J, L, O, P, R, U, V, X, Aa, Ab) 0.5 mm, (B, C, G, H, M, N, S, T, Y, Z) 2 mm, (K, Q, W, Ac) 3 cm.

We applied a forward genetic approach to isolate additional factors of the *SUB* signaling pathway, based on the hypothesis that mutations in some of the genes that are part of the *SUB* pathway should result in *sub*-like (*slm*) mutant phenotypes. We thus screened M2 families of an ethylmethane sulfonate-mutagenized L*er* population for *slm* mutants (see Materials and Methods). In this experiment we identified two new *sub* alleles [25] as well as several novel mutants with *sub*-like phenotypes (Figures 1–3). These fell into three different complementation groups, which map to distinct positions on chromosome 1 (Table 1). We termed two of the genes *DETORQUEO* (*DOQ*) and *ZERZAUST* (*ZET*), respectively. *DETORQUEO* refers to a latin term that means “to twist out of shape”. *ZERZAUST* is a German term for “disheveled”. We also isolated three mutant alleles of *QUIRKY* (*QKY*), which plays a role in fruit dehiscence (L.F., unpublished results; S.J. and Martin F. Yanofsky, unpublished observations) and the numbering of *qky* alleles was coordinated. Thus, our genetic approach resulted in the identification of three loci, *DOQ*, *QKY*, and *ZET*, mutations in which result in a *sub*-like phenotype and that, together with *SUB*, define the *STRUBBELIG-LIKE MUTANT* (*SLM*) class of genes.

**Figure 2 pgen-1000355-g002:**
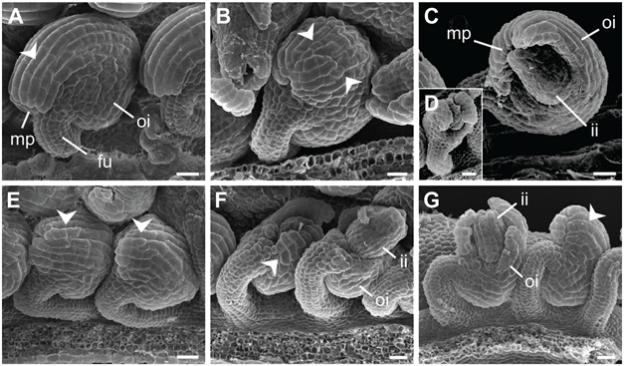
Comparison of ovule morphology in *sub-1*, *doq-1*, *qky-8*, and *zet-2* mutants. (A) Wild-type L*er*. The arrow marks one of the elongated cells of the distal outer integument. (B) *sub-1*. A mild phenotype is shown. Note the irregular size and shape of cells at the distal outer integument (arrow heads, compare to (A)). (C, D) *sub-1*. Strong phenotypes are depicted. Note the half-formed outer integument. (D) shows an example where the outer integument shows several gaps. (E) *doq-1*. This mutant shows a mild ovule phenotype comparable to the one depicted in (B). The arrow heads highlight the disruption of the regular cell files of the distal outer integument. (F) *qky-8*. Note the variability of the phenotype. The specimen to the left shows only a mild disorganisation of the cell shape (arrow head). The one to the right shows a strong phenotype (compare to (C)). (G) *zet-2*. This mutant shows a variable phenotype. The ovule to the left shows gaps in the outer integument while the one to the right exhibits only mild alterations in cell shape and size (arrow head). Abbreviations: fu, funiculus; ii, inner integument; mp, micropyle; oi, outer integument. Scale bars: 20 µm.

**Figure 3 pgen-1000355-g003:**
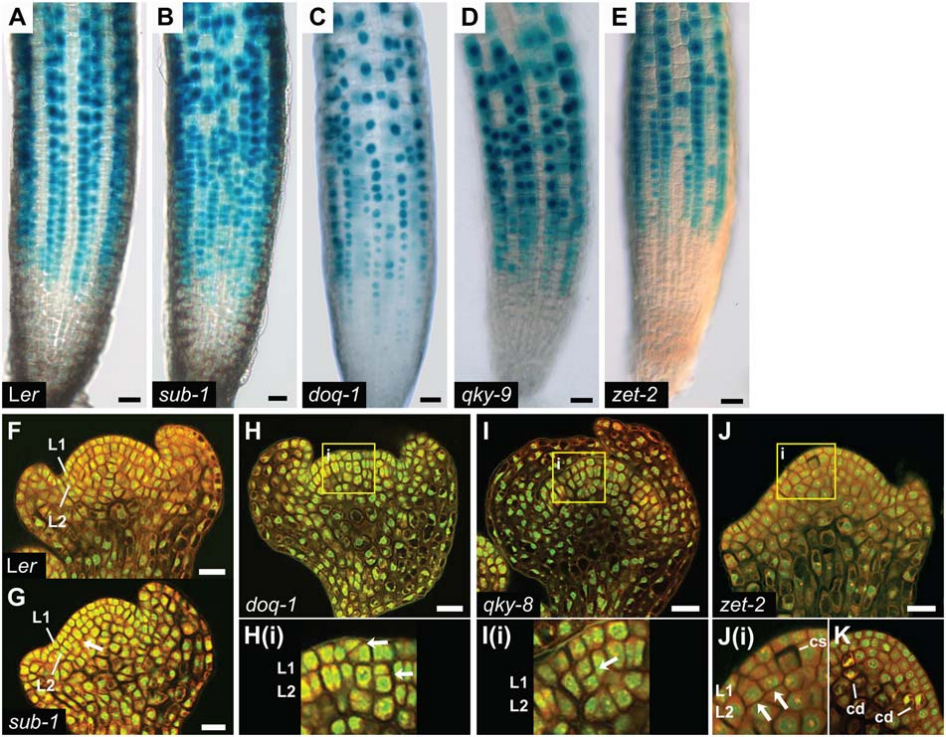
Analysis of cellular defects in 4-day old main roots and stage 3 floral meristems of *sub-1*, *doq-1*, *qky* and *zet-2* mutants. (A–E) Expression of the *GL2::GUS* reporter in whole-mount main roots. (A) Wild-type L*er* root. The reporter is detected in regular files of non-hair cells. (B–E) *GL2::GUS* reporter expression is patchy. (F–K) Mid-optical longitudinal sections through stage 3 floral meristems obtained by confocal microscopy of propidium-iodide-stained specimen. (F) Wild-type L*er*. Note the regular arrangement of cells in the L1 and L2. (G) *sub-1*. The arrow marks a periclinal cell division event. (H) *doq-1*. The region marked by the square (i) is shown at higher magnification in (Hi). The arrows highlight aberrant oblique and periclinal cell divisions in the L1 and L2, respectively. (I) *qky-8*. The region marked by the square (i) is shown at higher magnification in (Ii). The arrow labels a periclinal cell division in the L2. (J–K) *zet-2*. (J) The region marked by the square (i) is shown at higher magnification in (Ji). The arrows highlight periclinal cell divisions. A cell undergoing cell separation is indicated. (K) Disintegrating cells are marked. Abbreviations: cd, cell disintegration; cs, cell separation; L1, L1 cell layer; L2, L2 cell layer. Scale bars: (A–E) 25 µm, (F–K) 20 µm.

**Table 1:** Summary of *sub*-like mutants identified.

Mutant	Gene Symbol	*slm* identifier	Chromosome	Boundary markers	Map interval (AGI)*
*detorqueo-1*	*DOQ*	*slm8*	1	F10O3(48ID)^1^	0.75 Mb–3.21 Mb
				NF21M12^2^	
*zerzaust-1*	*ZET*	*slm26*	1	CER453151(58ID)^1^	22.29 Mb–24.13 Mb
				F13011(164ID)^1^	
*zerzaust-2*		*slm72*			
*quirky-7*	*QKY*	*slm3*	1	27.99(*Rsa*I)^1^	27.99 Mb–28.09 Mb
				F25A4(*Bgl*II)^1^	
*quirky-8*		*slm17*			
*quirky-9*		*slm38*			

*: Values represent approximate AGI positions of molecular markers listed.

1 Developed within this study.

2 Source: TAIR database and attributions therein.

### Comparison of *sub-1*, *doq-1*, *zet-2*, and *qky-8* Phenotypes

We identified one mutant allele of *DOQ* and two and three independent alleles of *ZET* and *QKY*, respectively (Table 1). All *sub*, *doq*, *zet* and *qky* alleles were recessive and behaved in a Mendelian fashion (not shown). The various *zet* and *qky* mutants did not noticeably differ in their respective phenotypes and the three *qky* alleles are likely to be nulls (see below). Thus, *zet-2* and *qky-8* or *qky-9* were used as reference alleles for further analysis. In addition, we used the well-characterised *sub-1* mutant for comparison [25]. This mutation likely represents a null-allele since it results in a stop codon and a predicted shorter SUB protein lacking the transmembrane and intracellular kinase domains. Thus, it is expected that *SUB*-dependent signaling across the plasma membrane is blocked in *sub-1* mutants.

At the macroscopic level *sub-1* mutants are known to be affected in several above-ground organs [25] (Figures 1–4) (Tables 2, 3). Inflorescences are characterised by reduced height, an irregularly twisted stem, and an aberrant phyllotaxis of flowers. Flowers open prematurely and show a large percentage of twisted and often notched petals. Furthermore, all flowers exhibit twisted carpels and about 70 percent of *sub-1* ovules showed aberrant initiation of the outer integument (Figure 2) (Table 2). This results in outer integuments with gaps that often resemble “multifingered clamps” or “scoops”. Also ovules with a fully developed outer integument show defects. In particular the distal or micropylar cells of the outer integument can show aberrant size and shape. In addition, about 40 percent of *sub-1* plants show at least one leaf per rosette with twisted petioles.

**Figure 4:**
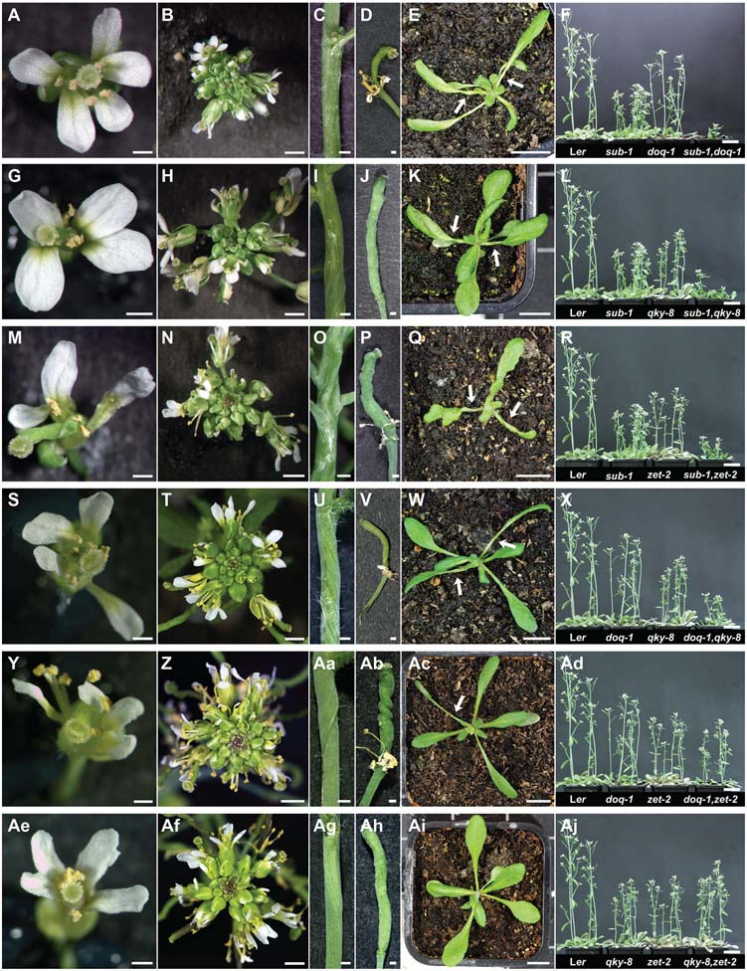
Comparison of above-ground morphology of *sub-1*, *doq-1*, *qky-8*, and *zet-2* double mutants. The given stages are identical across panels. (A–F) *sub-1 doq-1*. (G–L) *sub-1 qky-8*. (M–R) *sub-1 zet-2*. (S–X) *doq-1 qky-8*. (Y-Ad) *doq-1 zet-2*. (Ae–Aj) *qky-8 zet-2*. (A, G, M, S, Y, Ae) An open stage 13 flower from a 30-day old plant. Note the petal misorientation due to twisting along the longitudinal axis of petals. (M) *sub-1 zet-2*, (S) *doq-1 qky-8* and (Y) *doq-1 qky-8* flowers show particularly aberrant perianth morphology, with thin petals and heavy notching evident. (B, H, N, T, Z, Af) Top view of a 30-day inflorescence. (B) *sub-1 doq-1*, (H) *sub-1 qky-8* and (N) *sub-1 zet-2* inflorescences show aberrant phyllotaxy. (B, H, N, Z) Premature opening of flower buds is apparent. (C, I, O, U, Aa, Ag) A lateral view of a stem segment from a 30-day plant. (D, J, P, V, Ab, Ah) Morphology of a mature silique. Stem and silique twisting is exaggerated as compared to single mutant parentals, with the exception of the *qky-8 zet-2* double mutant (Ag, Ah). (D, P, V, Ab) Note floral organs have not abscised. (E, K, Q, W, Ac, Ai) Top view of a 16-day rosette. Arrows indicate exaggerated petiole twisting in leaves. (Ai) *qky-8 zet-2* leaf petioles are not twisted. (F, L, R, X, Ad, Aj) Plant height comparisons between Ler (left), single mutant parental (middle) and double mutant (right) 30-day plants. (F) *sub-1 doq-1* and (L) *sub-1 qky-8* mutants are significantly dwarfed, whereas (R) *sub-1 qky-8* and (X) *doq-1 qky-8* plants exhibit modest reductions in height. (Ad, Aj) *doq-1 zet-2* and *qky-8 zet-2* plant height is not significantly different from parental mutants. Scale bars: (A, C, D, G, I, J, M, O, P, S, U, V, Y, Aa, Ab, Ae, Ag, Ah) 0.5 mm, (B, H, N, T, Z, Af) 2 mm, (E, K, Q, W, Ac, Ai) 1 cm, (F, L, R, X, Ad, Aj) 3 cm.

**Table 2:** Ovule defects observed in *sub* and *slm* single and double mutants.

Genotype	Ovule Defect					Samples (N)
	Enclosed structure	<50% oi absent	≥50% oi absent	Severe malformation	Defective ii	
*sub-1*	60.4%	21.3%	16.8%	0%	0%	197
*doq-1*	96.8%	3.2%	0%	0%	0%	188
*qky-8*	69.3%	16.8%	13.9%	0%	0%	137
*zet-2*	52.4%	24.2%	23.4%	0%	<1%	124
*sub-1 doq-1*	39.1%	45.3%	15.6%	0%	10.9%	64
*sub-1 qky-8*	41.6%	13.2%	40.3%	4.9%	12.5%	144
*sub-1 zet-2*	28.6%	22.0%	28.6%	20.9%	17.6%	91
*doq-1 qky-8*	39.8%	39.8%	20.4%	0%	9.4%	191
*doq-1 zet-2*	27.3%	44.7%	24.0%	4.0%	13.3%	150
*qky-8 zet-2*	50.0%	35.6%	14.4%	0%	1.9%	216

oi, outer integument; ii, inner integument.

**Table 3:** Cellular defects in *sub* and *sub*-like mutant floral meristems.

	Periclinal cell division defects		Other cellular defects		
Mutant	L1	L2	CW sep.^1^	Cell dis.^2^	Samples (N)
L*er*	0%	2.6%	0%	0%	38
*sub-1*	3.3%	46.7%	0%	0%	30
*doq-1*	28.6%	64.5%	0%	0%	31
*qky-7*	4.0%	52.5%	0%	0%	25
*zet-2*	0%	43.7%	24.2%	18.7%	64

1 Cell wall separation.

2 Cell disintegration.

At the cellular level *sub-1* exhibits aberrant cell shape and robustly scorable numbers of periclinal, rather than anticlinal cell division planes in cells of the L2 layer of stage 3 floral meristems [25] (Figure 3) (Table 3). Interestingly, in this analysis we could also observe a previously unnoticed low number of periclinal divisions in the L1 of *sub-1* floral meristems. Furthermore, *sub*/*scm* mutants develop root hairs at discordant positions in the epidermis [26],[27]. This defect in root hair patterning can be followed by expressing the bacterial β-glucuronidase gene under the control of the *Arabidopsis GLABRA2* (*GL2*) promoter (*GL2::GUS*) [27],[33] (Figure 3). This reporter conveniently labels the regular files of non-hair cells in the epidermis of wild-type roots and exhibits an irregular expression pattern in *sub*/*scm* mutants.

Upon examination, many aspects of the phenotypes of *sub-1* and the other *slm* mutants were comparable. The *doq-1*, *qky-8* and *zet-2* mutants showed aberrant floral phyllotaxis and flowers with twisted petals (Figure 1). In addition, we observed premature floral bud opening in *doq-1* and *zet-2*. Petals of *zet-2* mutants showed notches similar to *sub-1* petals but *zet-2* floral organs were generally more misshapen. In rosettes, nearly all plants showed examples of leaf petiole twisting. In particular, *qky-8* mutants showed leaf twisting comparable to *sub-1*, whereas *doq-1* rosette leaves showed in addition elongated petioles and narrow blades. In contrast, *zet-2* rosette leaves did not show major defects at the gross morphology level. Irregular twisting of siliques and stems was apparent in *doq-1*, *qky-8* and *zet-2* mutants, although the twisting in *doq-1* siliques and stems was more subtle. Plant height was most affected in *qky-8* while *doq-1* and *zet-2* showed only a slight reduction. Mature ovules of *doq-1* mutants were mildly but consistently affected (Figure 2) (Table 2). The outer integument did not fully extend to the funiculus (reduced campylotropy). In addition, its distal cells were shorter and of irregular shape. Mature ovules of *qky-8* and *zet-2* closely resembled ovules of *sub-1* mutants with respect to gaps and altered cell shape in the outer integument. Interestingly, *zet-2* showed a slightly higher percentage of malformed outer integuments. As was the case for *sub* [25] the inner integument in all other *slm* single mutants appeared to be unaffected.

Further, *doq-1*, *qky-9* and *zet-2* mutants were investigated for cellular defects in the root epidermis and in L1/L2 cells of stage 2 to 4 floral meristems (Figure 3) (Table 3). In 4 day old main roots, all three mutants showed misregulation of *GL2::GUS* reporter expression comparable to *sub-1*, indicating that root hair patterning was similarly affected. The three mutants also exhibited cell shape and periclinal cell division plane defects in the L1 and L2 cells of floral meristems. Floral meristems of *doq-1* showed a higher percentage of those defects while in *zet-2*, cell separation and disintegration could also be observed. The latter finding indicates that *ZET* is also required for cell viability.

Taken together the analysis of the *slm* single mutants revealed that there is a large functional overlap of the corresponding genes and that individual *SLM* genes participate in subsets of *SUB*-dependent processes, such as ovule development, cellular behavior of L2 cells of stage 3 floral meristems, and root hair patterning. The results also suggest, however, that *SLM* genes have additional functions unrelated to each other. For example, *ZET* has a particular function in cell survival in floral meristems as has *DOQ* in the regulation of leaf shape.

### Double Mutant Analysis

**Figure 5:**
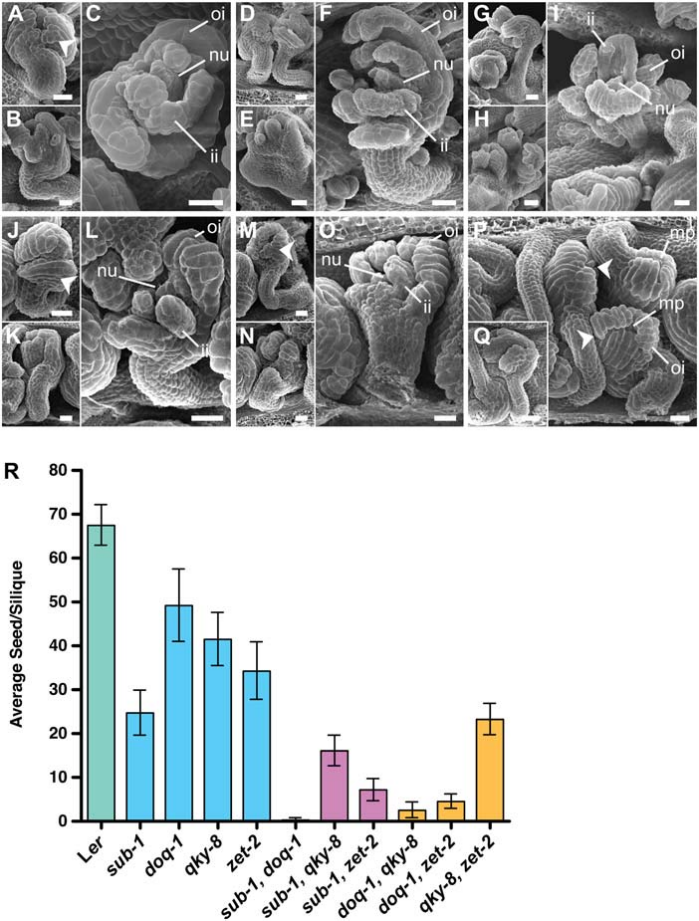
Comparison of ovule morphology in different *slm* double mutants. SEM micrographs of stage-4-V ovules are depicted. (A–C) *sub-1 doq-1*. (D–F) *sub-1 qky-8*. (G–I) *sub-1 zet-2*. (J–L) *doq-1 qky-8*. (M–O) *doq-1 zet-2*. (P,Q) *qky-8 zet-2*. Each double mutant shows a range of ovule phenotypes. (A, D, J, M and P) Mild phenotypes are depicted. Note the irregular size and shape of cells at the distal outer integument (arrow heads). (B, C, D, G K, N Q) Stronger ovule phenotypes are observed whereby specimens have half formed outer integuments. (C, F, I, L, O) In extreme cases, *sub-1 doq-1*, *sub-1 qky-8*, *sub-1 zet-2*, *doq-1 qky-8* and *doq-1 zet-2* ovules can show malformation of the inner integument. (C) This specimen is an example where the inner integument shows several gaps. (F, I, L, O) Inner integuments can also appear as finger-like protrusions. The *qky-8 zet-2* double mutant is an exception, whereby inner integument defects are rarely observed. (E, H) Severely malformed ovules can also appear as integument-like and nucellus-like structures emerging from placental tissue. (R) Fertility was assayed as the average seed number per silique (n = 20). Abbreviations: fu, funiculus; ii, inner integument; mp, micropyle; oi, outer integument. Scale bars: 20 µm.

To investigate further the genetic relationship between *SLM* genes we generated all possible double-mutant combinations and analysed the respective mutant phenotypes. The results are summarized in Figures 4 and 5 and Table 2.

### *sub-1 doq-1*

Petals of *sub-1 doq-1* double mutants mostly resembled *doq-1* petals (Figure 4A), however, stem twisting was more similar to *sub-1*. Other aspects of the *sub-1 doq-1* phenotype, such as silique twisting, plant dwarfism and rosette leaf petiole twisting were more exaggerated when compared to either single mutant. Ovules of *sub-1 doq-1* plants showed an increase in outer integument defects, although not as strong as in some other double mutant combinations. In addition, about 11% of ovules of *sub-1 doq-1* plants showed defects in inner integument morphology (Figure 5C) (Table 2), with gaps of variable sizes and finger-like protrusions. The fertility of *sub-1 doq-1* plants was severely reduced (Figure 5R).

### *sub-1 qky-8*

Petal and carpel twisting in *sub-l qky-8* double mutants was similar to that shown by each single mutant (Figure 4G). In contrast, twisting of siliques, stems, and leaf petioles was more pronounced as was the reduction in plant height (Figure 4I–L). In addition, ovule development was more heavily affected compared to the single mutants (Figure 5D–F) (Table 2), with 13% of ovules showing inner integument defects and a corresponding reduction in fertility (Figure 5R). About 5% of ovules were hardly recognizable as such, but rather resembled a mass of cells with integument-like outgrowths (Figure 5E).

### *sub-1 zet-2*

Overall, the *sub-1 zet-2* double mutants showed the most disturbed morphology (Figure 4M–R). Perianth organs were twisted, narrower and notched, while carpels were heavily twisted. As a result the overall structure of the flower was irregular. The stem phenotype was knotted rather than twisted. Leaf morphology was misshapen with more pronounced leaf petiole twisting and plants showed prominent dwarfism. Consistent with this exaggerated phenotype ovules of *sub-1 zet-2* plants showed severe defects with 21% of ovules resembling a mass of cells with integument-like outgrowths (Figure 5H) (Table 2). About 18% of *sub-1 zet-2* ovules exhibited short gapped outer integuments and similarly malformed inner integuments (Figure 5I). Again, fertility was reduced in *sub-1 zet-2* plants (Figure 5R).

### *doq-1 qky-8*

Perianth organs and carpels in *doq-1 qky-8* mutants were more twisted as compared to the parental lines (Figure 4S). Twisting of leaf petioles, stems, and siliques was also more pronounced as was dwarfism. Ovules of *doq-1 qky-8* mutants showed a less exaggerated phenotype compared to some of the other double mutant combinations. Still, a large proportion of *doq-1 qky-8* ovules showed gaps in the outer integument (Figure 5J–L) (Table 2) and aberrant inner integument morphology was seen in 9% of ovules (Figure 5L). Fertility was strongly reduced in *doq-1 qky-8* double mutants (Figure 5R).

### *doq-1 zet-2*

Perianth morphology in *doq-1 zet-2* mutants was about equivalent to that observed in *sub-1 zet-2* and *doq-1 qky-8* flowers (Figure 4Y). Twisting of stems appeared slightly more pronounced compared to each single mutant while siliques were drastically more twisted. Plant height was comparable to *zet-2* single mutants. Overall a higher percentage of *doq-1 zet-2* ovules showed gaps in the outer and inner integuments (Figure 5M–O), with 4% of ovules exhibiting severe malformations comparable to *sub-1 zet-2* mutants (Table 2). The reduction in fertility was comparable to that of *doq-1 qky-8* double mutants (Figure 5R).

### *qky-8 zet-2*

The *qky-8 zet-2* double mutants largely phenocopied *zet-2* single mutants in above-ground morphology (Figure 4Ae–Aj) including ovules (Figure 5P,Q) (Table 2). One exception to this was leaf petiole twisting as this aspect was most similar to the phenotype in *qky-8* single mutants.

In summary, the pleiotropic phenotypes of *slm* single and double mutants complicated the double mutant analysis. Although an exaggerated phenotype was often observed in a double mutant combination it was usually difficult to decide whether a double mutant displayed an additive or synergistic phenotype, or whether a particular mutation was epistatic to another. Overall, the double mutant analysis reinforced the notion that *SLM* genes likely do not act in a single linear pathway but have both overlapping and separate functions.

### Phenotypic Analysis of *doq*, *qky*, and *zet* Plants Carrying a *35S::SUB* Transgene

**Figure 6:**
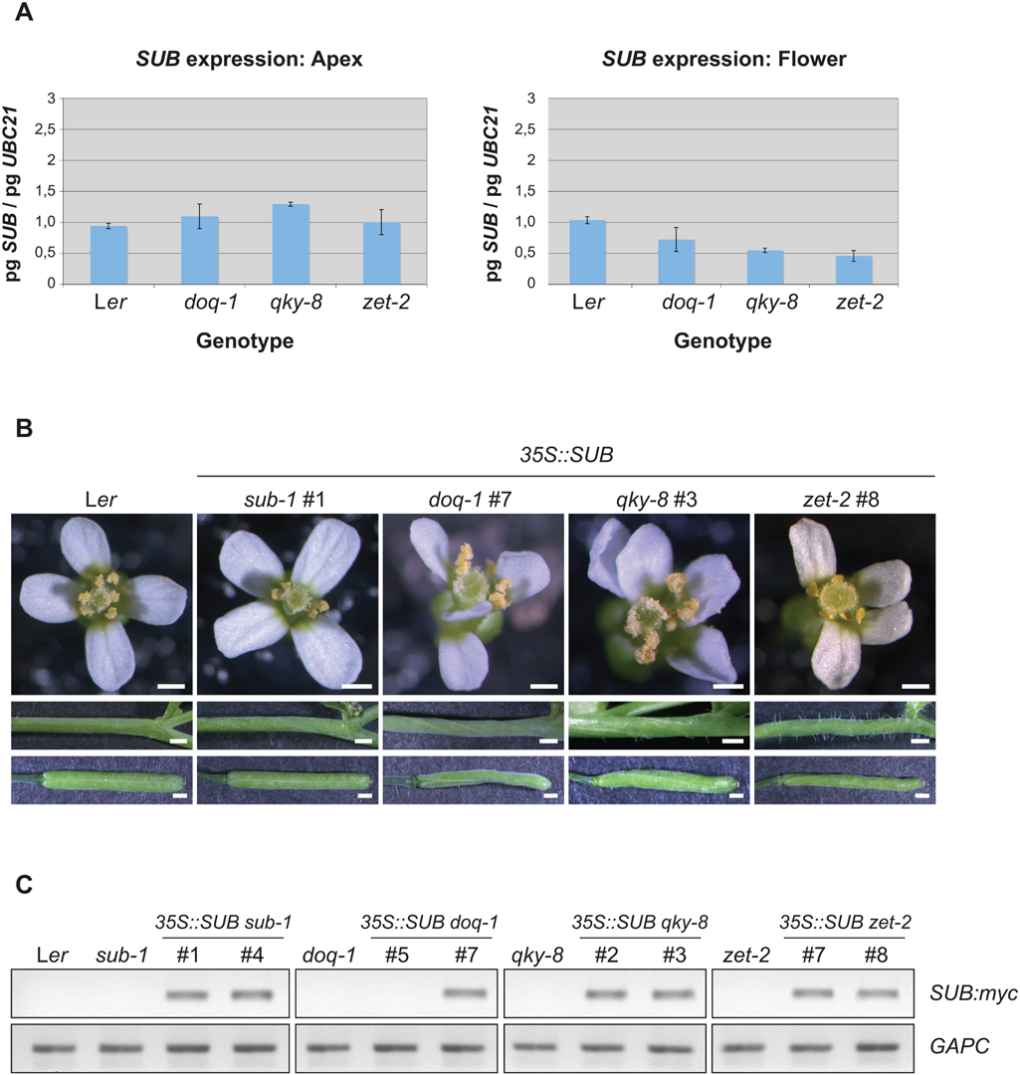
Analysis of *SUB* expression in the *slm* plant lines. (A) Quantitative expression analysis of *SUB* in the *slm* mutants. Steady-state mRNA levels were measured in apex and flower tissues by quantitative real-time PCR. *UBC21* expression was used for normalization. (B) Phenotypes of *35S:SUB slm* transgenic lines. A *35S::SUB:myc* construct was transformed into each *slm* mutant background. T1 plants were analysed for rescue of flower, silique and stem mutant phenotypes. Note only *sub-1* mutants exhibit rescue by *SUB* overexpression. (C) Transgenic *35S:SUB:myc* T1 plants represented in (A) were assayed for *SUB* transgene expression using semi-quantitative RT-PCR (top panels). *GAPC* [118] served as internal control (bottom panels). Scale bars: 0.5 mm.

To further explore the genetic relationship between *SLM* genes we first tested whether *SUB* expression was responsive to other genes of this group. We investigated *SUB* expression in inflorescence apices and stage 10–12 flowers (see below) from several *slm* mutants by quantitative real time PCR (qRT-PCR). As can be seen in Figure 6A only very moderate changes in *SUB* expression were detected with a slight but consistent reduction of *SUB* expression in flowers of *doq-1*, *qky-8* and *zet-2* mutants. In contrast, *SUB* transcript levels were unaltered in those tissues and mutants when assessed by a transcriptome analysis (see below). Given that *SUB* is expressed at very low levels to begin with [34] we reasoned that the observed mild effects may not be the result of direct effects but rather be a consequence of indirect influences due to tissue sampling or the altered morphology of the mutants. To test the relevance of the effects we asked whether rendering *SUB* independent of its normal transcriptional regulation, by ectopically expressing *SUB* using the cauliflower mosaic virus 35S promoter, could result in phenotypic rescue of *doq*, *qky* and *zet* mutants. We analysed the phenotypes of *35S::SUB doq-1*, *35S::SUB qky-8* and *35S::SUB zet-2* plants (Figure 6B). In particular we scored stem twisting, flower morphology and silique twisting of individual transgenic T1 lines. Transgene functionality was demonstrated by the wild-type appearance of *35S::SUB sub-1* plants. Despite demonstrating comparable transgene expression (Figure 6C), the other tested combinations did not show phenotypic rescue (*35S::SUB doq-1*: 42 T1 lines scored, *35S::SUB qky-8*: >100, and *35S::SUB zet-2*: >100), indicating that *SUB* does not act as a downstream target gene of *DOQ*, *QKY* or *ZET*. Taken together our data suggest that *SUB* is not directly regulated at the transcriptional level by the other *SLM* genes.

### Genome-Wide Transcriptome Analysis and Global Expression Profiling

To shed light on the molecular nature of *SUB*-dependent processes and to systematically investigate the similarities between the *SLM* genes at the mechanistic level, we applied a transcriptome analysis. Since mutants with defects in genes of overlapping functions should also exhibit overlapping alterations in transcript profiles we compared wild type (L*er*) with plants of two *sub* alleles , as well as *doq-1*, *zet-2* and *qky-8* mutants using the Affymetrix ATH1 GeneChip platform (see Materials and Methods). The analysis of two independent *sub* alleles was aimed at obtaining a robust set of *SUB*-responsive genes for the different pairwise comparisons and the list of *SUB*-responsive genes represents the overlap of misexpressed genes in *sub-1* and *sub-3* mutants [25]. Since we had observed tissue-specific differences in the phenotypes of *slm* mutants, which suggested tissue-specific sub-functions for the corresponding genes, we tried to capture these differences also in the sampling for transcriptome analysis. To this end, we sampled inflorescence apices plus flowers up to stage 9 (apex data set), representing mostly proliferative tissues, while stage 10–12 flowers were collected to represent maturation stages (flower data set). The samples thus covered *SUB*-related aspects such as the control of cell shape/division plane in L2 cells of floral stage 3 meristems, and the regulation of petal, carpel and ovule development.

Since traditional analysis tools to identify differential gene expression in whole genome transcriptome data are not well suited for comparisons of multiple samples we developed a meta analysis tool based on Z-score statistics (see Materials and Methods). It offers a variety of benefits for the simultaneous analysis of multiple pairwise comparisons, such as normalization for the overall biological effect observed in the individual experiments, increased sensitivity, and the possibility of querying data using Bolean logic. Using this tool we identified 89 and 193 significantly misexpressed genes, respectively, in the apex and flower samples of *sub* plants (Table 4). The other mutants were characterised by higher numbers of misexpressed genes (Table 4) indicating that *DOQ*, *QKY*, and *ZET* affect more processes than *SUB*. More importantly, we systematically analyzed the overlap of misexpressed genes from individual *slm* mutants compared to wild type. The results of all pairwise comparisons are given in Table 5. For example, a pairwise comparison between *sub* and *doq-1* in the apex sample revealed a 42 gene overlap. This corresponded to 47% of genes misexpressed in *sub* and about 17% of genes aberrantly expressed in *doq-1*. Comparable values were observed for *sub* versus *qky-8* and *sub* vs *zet-2* comparisons. The overlap with *SUB*-dependent genes was even more pronounced in the flower data set and in the *sub*-*doq-1* comparison we found 118 common genes, representing 61% and 17% of the genes misexpressed in *sub* and *doq-1*, respectively. The *sub qky-8* and *sub zet-2* comparisons revealed higher values with 76% and 81% of *SUB*-responsive genes being found to overlap. Thus, a significant number of *SUB*-responsive genes are also sensitive to *DOQ*, *QKY* and *ZET* function. Similar pairwise comparisons were made between *doq-1*, *qky-8* and *zet-2* (Table 6). Again there was considerable overlap of misexpressed genes between different mutants. Most strikingly, in stage 10–12 flowers 67% and 71% of the *QKY*-responsive genes are also misregulated in *doq-1* and *zet-2*, respectively. This finding implies that the activity of more than two-thirds of *QKY*-responsive genes also depends on *DOQ* and *ZET* function in stage 10–12 flowers. Taken together the microarray data corroborate the morphological analysis of the single mutants and are compatible with the notion that the different *SLM* genes share overlapping functions.

**Table 4:** Genes misexpressed in *sub* and *slm* mutants.

Mutant profiles (Apex)	Genes^1^	Mutant profiles (Flower)	Genes^2^
*sub*	89	*sub*	193
*doq-1*	255	*doq-1*	698
*qky-8*	158	*qky-8*	352
*zet-2*	330	*zet-2*	497

1 Genes misexpressed in apex tissues sampled (up to flower stage 9).

2 Genes misexpressed in flowers (stages 10–12).

**Table 5:** Comparison of genes co-misexpressed in *sub* and *slm* mutants.

Apex Overlap profiles^1^	Genes	% *sub* 2	% *slm* 3	Flower Overlap profiles^1^	Genes	% *sub* 2	% slm^3^
*sub_ doq-1*	42	47.2%	16.5%	*sub_ doq-1*	118	61.1%	16.9%
*sub_ qky-8*	40	44.9%	25.3%	*sub_ qky-8*	147	76.2%	41%
*sub_ zet-2*	48	53.9%	14.5%	*sub_ zet-2*	156	80.8%	31.4%

1 Denotes a comparison (overlap profile) made between two mutant transcriptome profiles.

2 Percentage of *sub* misexpressed genes.

3 Percentage of *slm* misexpressed genes represented in listed comparisons.

Percentages are calculated based on mutant gene list totals (Table 4).

**Table 6:** Comparisons of genes co-misexpressed in *slm* mutants.

Apex Overlap profiles^1^	Genes	% *slm* 2	% *slm* 3	Flower Overlap profiles^1^	Genes	% *slm* 2	% *slm* 3
*doq-1_ qky-8*	66	25.9%	41.8%	*doq-1_ qky-8*	237	34.0%	67.3%
*doq-1_ zet-2*	86	33.7%	26.1%	*doq-1_ zet-2*	250	35.8%	50.3%
*qky-8_ zet-2*	63	39.9%	19.1%	*qky-8_ zet-2*	248	70.5%	49.9%

1 Denotes a comparison (overlap profile) made between two mutant transcriptome profiles.

2,3 Percentage of *slm* misexpressed genes (^2^first appearing, ^3^second appearing) in listed comparisons.

Percentages are calculated based on mutant gene list totals (Table 4).

**Figure 7:**
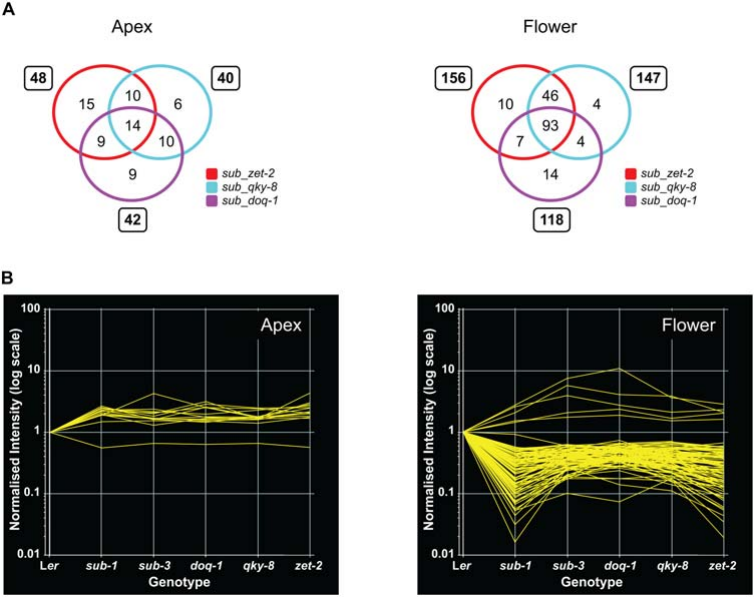
Comparison of gene expression changes between *sub* and *slm* mutants. (A) Venn diagrams showing the overlap of genes detected as significantly misexpressed (z-score<−2 or >2) in *sub*, *doq-1*, *qky-8* and *zet-2* mutants. Analyses were performed using data generated from apex (left) and late-stage flower (right) probe sets. Gene lists that compared *sub* to each independent *slm* mutant were initially generated (numbers in boxes) and then overlapped for a final comparison (quadruple overlap set). (B) Line graphs depicting the expression profiles of genes present in the quadruple overlap set across *sub* and *slm* mutants in apex (left) and flower (right) tissues. Note how the up- or downregulation of individual genes coincides across all mutant genotypes.

**Figure 8:**
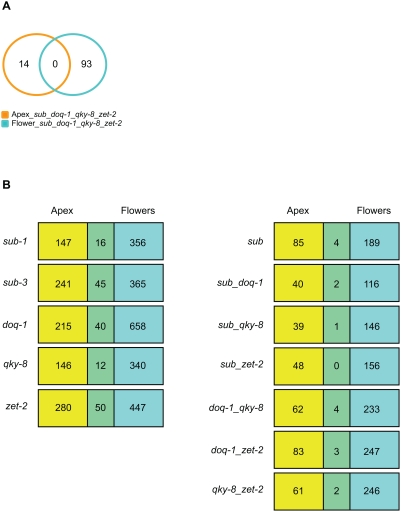
Comparison of genes misexpressed in apex and flower overlap sets. (A) Venn diagram depicting the overlap of genes misexpressed in apex and flower quadruple overlap sets. (B) Box diagram detailing the overlap of genes misregulated in apex and flower gene sets for individual *sub* and *slm* comparisons.

To further study the complexity of the functional relationship among *SLM* genes we carried out additional pairwise comparisons, examined the identity of genes present in the overlap lists, and identified transcripts which are misregulated in all four mutants (i.e., a *sub*, *doq-1*, *qky-8* and *zet-2* “quadruple” overlay) (Figures 7,8; Tables 5,6; Datasets S1, S2). While doing so we uncovered striking trends in the transcriptome data. For example, we found that more than 30% of the misregulated genes found in any *sub*/*slm* overlap identified in the apex samples are indeed shared by all mutants (Figure 7A). More significantly, 13 of the 14 transcripts identified as common *SLM* responsive in the apex were consistently elevated in expression in all mutants, suggesting that a core function of the *SLM* genes is to repress this set of genes in proliferating tissue (Figure 7B). Strikingly, this trend was reversed in differentiating tissue represented by the floral samples. Here the misregulated genes shared by all mutants made up more than 65% of transcripts identified in pairwise comparisons (Figure 7A) and 88 of the 93 genes were reduced in expression (Figure 7B). In addition to supporting the notion of functional overlap between the *SLM* genes, this analysis also demonstrated that diversification of *SLM* function observed in different tissues is based on a dramatic switch in the underlying molecular mechanism. While signaling through *SLM* proteins in proliferating tissue mainly causes repression of target gene transcription, *SLM* mediated signals lead to a transcriptional activation of downstream genes in differentiating cells, suggesting that SUB and other SLM proteins interact with a radically different regulatory environment. This notion is supported by another striking observation: while we found strong similarities between different genotypes within one tissue we observed very little overlap in misregulated genes between the same genotype in the two tissues analysed (Figure 8). For example, only four misregulated transcripts were shared in apex and flower samples of *sub* mutants. Similar observations were made for the other single and pairwise comparisons. In addition, the core target gene sets of apex and flower, derived from quadruple comparisons, did not share any transcripts.

To investigate the nature of *SLM*-dependent processes we assessed the known or predicted functions of the apex and flower core sets of *SLM*-responsive genes by searching The Arabidopsis Information Resource (TAIR), the literature and making use of the AtGenExpress expression atlas [34] with the help of the AtGenExpress Visualisation Tool (AVT, http://jsp.weigelworld.org/expviz/) (Tables 7, 8). In both core sets we observed a strong representation of genes that are involved in processes, such as cell wall biosynthesis and function, hormone signaling and abiotic and biotic stress responses. For example, two genes in the apex core set encode the inositol oxygenase family enzymes MIOX2 and MIOX4 required for biosynthesis of uridine-diphospho-glucuronic acid (UDP-GlcA), a precursor for various cell-wall matrix polysaccharides [35]. In addition, *GERMIN-LIKE PROTEIN 6* (*GLP6*) is a member of a gene family encoding putative extracellular GERMIN-LIKE proteins [36] some of which play a role in biotic stress responses and cell wall biology. The flower core set of *SLM*-responsive genes also included several genes coding for cell wall proteins, such as *ECS1* [37], *At2g05540* (Glycine-rich protein), *At5g03350* and *At5g18470* (lectin family proteins), and *At2g43570* and *At4g01700* (chitinases). In another example that relates to hormone signaling, five of the 13 upregulated apex core set genes were inducible by jasmonates (JA) while the flower core set was characterized by a large group of genes that relate to different aspects of signaling mediated by salicylic acid (SA) (see Discussion). *SLM*-responsive genes whose expression is sensitive to JA encoded for example JAZ1, a central negative regulator of JA signaling [38],[39], ERD5 a mitochondrial proline dehydrogenase (ProDH) [40], AtLEA5/SAG21 [41],[42], and AtTTPG, a class III trehalose-6-phosphate phosphatase involved in the biosynthesis of trehalose [43],[44]. All of these genes are believed to contribute to the plant's response to dehydration, cold and oxidative stress [40], [41], [44]–[50]. We also observed *SLM*-dependency of genes involved in the homeostasis of the auxin indole-3-acetic acid (IAA) and the production of the *Arabidopsis* phytoalexin camalexin and indole glucosinolates. Glucosinolate metabolites play important roles in plant nutrition, growth regulation and the interactions of plants with pathogens and insect herbivores [51],[52]. In the apex core set we identified *IAA-LEUCINE RESISTANT-LIKE GENE 6* (*ILL6*), which belongs to a gene family encoding IAA amino acid conjugate hydrolases involved in the release of the active auxin from corresponding inactive amino acid conjugates [53],[54]. *CYP83B1* and *CYP79B2* encode cytochrome P450 monooxygenases that regulate the pool of indole-3-acetaldoxime (IAOx) [55]–[58] a key branching point of primary and secondary metabolic pathways and a precursor of indole glucosinolates, camalexin [59], and IAA (reviewed in [51],[52]). In addition, the flower core set contained two downregulated cytochrome P450 genes, *CYP71A13* and *CYP71B15*, which promote distinct steps in camalexin biosynthesis [60]–[63].

**Table 7:** List of genes co-misexpressed in *slm* mutant apex tissues.

Gene/Keyword	Gene Identifier	Description^1^
**Genes upregulated**		
*MIOX2*	At2g19800	Myo-inositol oxygenase family gene / biosynthesis of nucleotide sugar precursors for cell-wall matrix
*MIOX4*	At4g26260	Myo-inositol oxygenase family gene / biosynthesis of nucleotide sugar precursors for cell-wall matrix
*GLP6*	At5g39100	Germin-like family protein / cell wall biology, pathogen response, nutrient reservoir
*JAZ1*	At1g19180	JASMONATE-ZIM-DOMAIN PROTEIN 1 / nuclear-localized protein negatively regulating jasmonate signaling, inducible by JA
*ERD5*	At3g30775	EARLY RESPONSIVE TO DEHYDRATION 5. Proline dehydrogenase / osmotic stress responsive, inducible by JA
*ATSIP2*	At3g57520	ARABIDOPSIS THALIANA SEED IMBIBITION 2 / glycosyl hydrolase, catabolism of raffinose family oligosaccharides, inducible by JA
*AtLEA5*	At4g02380	LATE embryogenesis abundant-like protein 5 / response to cold, oxidative stress, reactive oxygen species, inducible by JA
*AtTTPG*	At4g22590	Trehalose-6-phosphate phosphatase / trehalose biosynthetic process, sugar-based signaling, inducible by JA
*ILL6*	At1g44350	AA-LEUCINE RESISTANT (ILR)-LIKE GENE 6 / IAA-amino acid hydrolase
*CYP79B2*	At4g39950	Cytochrome P450 family/tryptophan metabolism / converts Trp to indo-3-acetaldoxime (IAOx), a precursor to IAA, camalexin and indole glucosinolates
*CYP83B1*	At4g31500	Cytochrome P450 monooxygenase / biosynthetic pathway of indole glucosinolates
Nodulin MtN21-like	At2g39510	Nodulin MtN21 family protein/function unknown.
Unknown	At1g74055	Unknown protein
**Genes downregulated**		
*AtDof5.8*	At5g66940	Dof-type zinc finger domain-containing protein / putative transcription factor, possibly involved in vascular development

Gene keywords and gene identifiers are shown.

1 Gene descriptions were derived from TAIR and attributions therein.

**Table 8:** List of genes co-misexpressed in *slm* mutant flowers.

Gene/Keyword^1^	Gene Identifier	Description^2^
**Genes upregulated**		
*ATHB40*	At4g36740	Homeodomain leucine zipper class I (HD-Zip I) protein/ transcription factor
*GLTP3*	At3g21260	GLYCOLIPID TRANSFER PROTEIN 3
Glycine-rich	At2g05540	Glycine-rich protein
Lactoylglutathione lyase	At1g15380	Lactoylglutathione lyase family protein
*SUS3*	At4g02280	sucrose synthase 3/putative sucrose synthase activity
**Genes downregulated**		
*AGP5*	At1g35230	Arabinogalactan-protein 5
*AIG1*	At1g33960	AVRRPT2-INDUCED GENE 1/ exhibits RPS2- and avrRpt2-dependent induction early after bacterial infection
*ANAC036*	At2g17040	Arabidopsis NAC domain containing protein 36/ transcription factor
*ATARD3*	At2g26400	acireductone dioxygenase (ARD/ARD')family protein 3, putative
*ATGH9B17*	At4g39000	ARABIDOPSIS THALIANA GLYCOSYL HYDROLASE 9B17/ O-glycosyl hydrolase activity
*ATNUDT6*	At2g04450	ARABIDOPSIS THALIANA NUDIX HYDROLASE HOMOLOG 6/ DNA metabolism, putative pathogen response protein
*COBL5*	At5g60950	COBRA-LIKE PROTEIN 5 PRECURSOR/ phytochelatin synthetase, putative
*CYP71A13*	At2g30770	CYTOCHROME P450 MONOOXYGENASE 71A13/IAA and camalexin biosynthetic process, pathogen defence response
*CYP71B15*	At3g26830	CYTOCHROME P450 FAMILY, 71B15/camalexin biosynthesis
*CYP96A2*	At4g32170	CYTOCHROME P450 FAMILY, 96A2
*ECS1*	At1g31580	ECOTYPE SPECIFIC 1/ plant cell wall-associated protein.
*EDS1*	At3g48090	ENHANCED DISEASE SUSCEPTIBILITY 1 / SA-mediated signaling, defense against pathogens
*EDS5*/*SID1*	At4g39030	ENHANCED DISEASE SUSCEPTIBILITY 5 / MATE-transporter family protein / SA biosynthesis, defense against pathogens
*EXP3*	At2g18660	Expansin family protein
ABA	At5g13200	GRAM domain-containing protein/ABA-responsive protein-related, function unknown
Ankyrin	At5g54610	Ankyrin repeat family protein
Auxin-response	At5g35735	Auxin-responsive family protein
Calcium-ion binding	At3g47480	Calcium-binding EF hand family protein
Calcium-ion binding	At5g39670	Calcium-binding EF hand family protein
Calcium-ion binding	At3g50770	Calmodulin-like protein 41, predicted calcium binding activity
Chitinase	At2g43570	Chitinase protein, putative
Chitinase	At4g01700	Chitinase protein, putative
DEDOL-PP	At5g58782	Dehydrodolichyl diphosphate synthase (DEDOL-PP), putative
DELTA-VPE	At3g20210	Delta vacuolar processing enzyme/seed coat development
Disease resistance	At1g57650	Disease resistance protein RPP1-WsA, putative
Disease resistance	At1g72900	Disease resistance protein (TIR-NBS class), putative
Disease resistance	At1g72920	Disease resistance protein (TIR-NBS class), putative
Glucosidase	At3g03640	Beta-glucosidase
*GRX480*	At1g28480	Glutaredoxin family protein 5/ regulates protein redox state, JA-mediated signaling pathway, SA-mediated signaling pathway
HIN1-related	At1g65690	Harpin-induced protein-related/HIN1-related protein
*ICS1*/*SID2*/*EDS16*	At1g74710	isochorismate synthase 1/ salicylic acid biosynthetic process
Lectin family	At5g03350	Legume lectin family protein, putative
Lectin family	At5g18470	Curculin-like (mannose-binding) lectin family protein
Lipase	At5g55050	GDSL-motif lipase/hydrolase family protein
Lipid transfer protein	At3g22600	Protease inhibitor/seed storage/lipid transfer protein (LTP) family protein
Lipid transfer protein	At5g55450	Protease inhibitor/seed storage/lipid transfer protein (LTP) family protein
LRR protein	At2g24160	Leucine rich repeat protein family, putative disease resistance protein
Methionine metabolism	At4g21850	Methionine sulfoxide reductase domain-containing protein
MIF protein	At3g51660	Macrophage migration inhibitory factor (MIF) family protein
*NIMIN1*	At1g02450	Transcriptional regulator/ negative regulator of SA-dependent signal transduction, modulates PR gene expression
*NRAMP5*	At4g18790	NRAMP METAL ION TRANSPORTER 5
Oxidoreductase	At5g24530	2OG-Fe(II) oxygenase family protein
*PBP1*	At5g54490	PINOID-BINDING PROTEIN 1/ responsive to auxin stimulus
*PBS3*/*GDG1*	At5g13320	AVRPPHB SUSCEPTIBLE 3/ Required for accumulation of salicylic acid, activation of defense responses and resistance
*PCC1*	At3g22231	PATHOGEN AND CIRCADIAN CONTROLLED 1/regulated by the circadian clock, responsive to pathogens
Plastocyanin domain	At4g30590	Plastocyanin-like domain-containing protein
*PR1*	At2g14610	PATHOGENESIS-RELATED GENE 1 / pathogen defense response, SAR, response to vitamin B1
*PR2*	At3g57260	PATHOGENESIS-RELATED GENE 2 / glycosyl hydrolase family 17 protein, cold responsive, SAR
*PR4*	At3g04720	PATHOGENESIS-RELATED GENE 4/ hevein-like protein precursor, responsive to virus and ethylene stimulus, SAR
*PR5*	At1g75040	PATHOGENESIS-RELATED GENE 5, thaumatin-like protein/ SAR, response to UV-B, regulation of anthocyanin biosynthetic process
Protease	At3g51330	Aspartyl protease family protein
Protein binding	At1g10340	Ankyrin repeat family protein
Protein binding	At4g14365	Zinc finger (C3HC4-type RING finger) family protein/ankyrin repeat family protein
Protein kinase	At4g11890	Protein kinase family protein
Protein kinase	At4g23150	Protein kinase family protein
Receptor	At2g37710	Receptor lectin kinase/ responsive to SA stimulus
*RLK1*	At5g60900	RECEPTOR-LIKE PROTEIN KINASE 1
*RLK6*	At4g23130	RECEPTOR-LIKE PROTEIN KINASE 6
*SAL2*	At5g64000	3′(2′),5′-bisphosphate nucleotidase
Self-incompatibility	At1g26795	Self-incompatibility protein-related
*SIB1*	At3g56710	SIGMA FACTOR BINDING PROTEIN 1
*TAT3*	At2g24850	TYROSINE AMINOTRANSFERASE 3 / responsive to wounding and JA treatment
Transporter	At3g10780	emp24/gp25L/p24 family protein / putative transporter protein
*UBC31*	At1g36340	UBIQUITIN-CONJUGATING ENZYME 31
*WAK1*	At1g21250	WALL ASSOCIATED KINASE 1 / response to salicylic acid stimulus
*WAKL8*	At1g16260	WALL ASSOCIATED KINASE-LIKE 8
*WRKY33*	At2g38470	WRKY-type DNA binding protein/ defence response to bacterium and fungus
*WRKY46*	At2g46400	WRKY-type DNA binding protein
*WRKY70*	At3g56400	WRKY-type DNA binding protein / transcriptional repressor, JA mediated signaling pathway, SA mediated signaling pathway, SAR
*WRKY53*	At4g23810	WRKY-type DNA binding protein/ positive regulation of transcription, regulation of defense response
*WRKY38*	At5g22570	WRKY-type DNA binding protein / defence response, SAR
*ZAT10*	At1g27730	Salt tolerant zinc finger / transcriptional repressor activity, cold, water deprivation, salt stress, wounding, ABA and chitin responsive
Unknown	At1g19020	Expressed protein
Unknown	At3g60420	Expressed protein
Unknown	At5g52975	Expressed protein
Unknown	At1g13470	Hypothetical protein
Unknown	At1g52970	Hypothetical protein
Unknown	At1g56060	Hypothetical protein
Unknown	At3g13950	Hypothetical protein
Unknown	At3g14850	Hypothetical protein
Unknown	At3g48640	Hypothetical protein
Unknown	At3g48650	Hypothetical protein, predicted pseudogene
Unknown	At4g23610	Hypothetical protein
Unknown	At5g19240	Hypothetical protein
Unknown	At2g18680	Unknown protein
Unknown	At2g14560	Unknown protein
Unknown	At1g65500	Unknown protein

1 Gene keywords and gene identifiers are shown.

2 Gene descriptions were derived from TAIR and attributions therein.

ABA, abscisic acid; DR, disease resistance; JA, jasmonic acid; PR, pathogenesis-related; SA, salicylic acid; SAR, systemic acquired resistance.

### Molecular Identification of *QKY*

**Figure 9:**
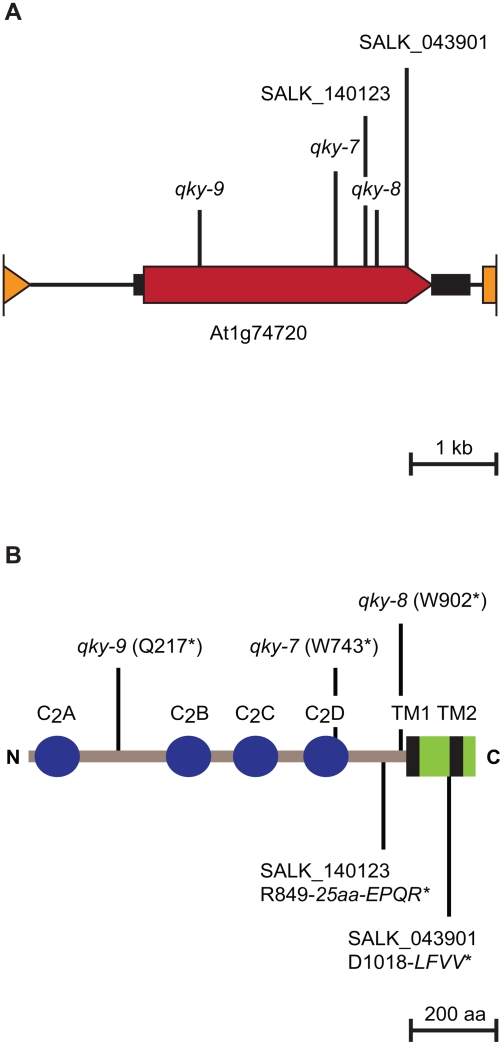
Molecular characterization of *QKY*. (A) Molecular organization of the *QKY* locus on chromosome 1. The horizontal black line highlights genomic DNA. The red arrow indicates the orientation and extent of the *QKY* open reading frame. The small black boxes mark the 5′ and 3′ untranslated regions, respectively. The orange arrow and box denote the end of the preceding and the start of the next annotated open reading frames, respectively. The positions of the various point mutations or T-DNA insertion sites are marked. (B) Predicted domain topology of the QKY protein. The sequence and numbering of the C_2_ domains, and the altered C-termini of the mutant protein variants, are given. The italic capital letters represent the de novo residues present in the predicted mutant QKY proteins from the two T-DNA lines. Stars mark the premature stops in the predicted mutant proteins. The green box denotes the plant phosphoribosyltransferase C-terminal region (PRT_C). Abbreviations: aa, amino acids; N, amino terminus; C, carboxy terminus; TM, transmembrane domain.

As a first step to molecularly define the *SLM* pathway, the *QKY* gene was identified by map-based cloning (Figure 9 and Materials and Methods). In all three EMS-induced *qky* alleles we could identify single point mutations in At1g74720 (Figure 9A). Further, two distinct T-DNA insertions in this locus result in *qky* mutants phenocopies (not shown). Combined this demonstrates that At1g74720 is *QKY*. Sequence analysis predicted that *QKY* contains no introns and encodes a transmembrane protein of 1081 amino acids with a calculated molecular mass of 121.4 kDa harboring four C_2_ domains (Figure 9B). In addition, two transmembrane domains are embedded in a plant-specific phosphoribosyltransferase C-terminal region (PRT_C, PFAM identifier PF08372), which according to the PFAM database occurs characteristically at the carboxy-terminus of phosphoribosyltransferases and phosphoribosyltransferase-like proteins [64]. Interestingly, this domain often appears together with C_2_-domains, as in QKY. A related domain topology, albeit with only three C_2_ domains, is found in several proteins present in humans, *Drosophila melanogaster* and *Caenorhabditis elegans* (*C. elegans*), collectively referred to as multiple C_2_ domain and transmembrane region proteins (MCTPs) [65]. The role of animal MCTPs is not well defined despite the fact that in *C. elegans* a single MCTP gene is present and essential for embryo viability [66]. *QKY* represents the first described plant MCTP-class gene. In accordance with proposed transmembrane topography for MCTPs the QKY C_2_ domains most likely have an intracellular localisation as QKY lacks a distinguishable N-terminal signal peptide and all known C_2_ domains are cytoplasmic. C_2_ domains are autonomously folding modules and form Ca^2+^-dependent phospholipid complexes, although some exceptions are known that do not bind Ca^2+^ or phospholipids or both [67]–[69]. Other animal proteins with multiple C_2_ domains, but only one transmembrane region, include the synaptotagmins [70], the extended synaptotagmins [71], and the ferlins [72]. Synaptotagmins and ferlins are known to play a role in membrane trafficking. Although there is general resemblance in domain topology, very little primary sequence conservation is observed between QKY and the animal MCTPs, synaptotagmins, and ferlins.

The *qky-7*, *qky-8*, and *qky-9* alleles likely cause a complete loss of *QKY* function. All three mutations are predicted to introduce stop codons leading to shorter proteins with variable numbers of C_2_ domains but always lacking the two transmembrane domains (Figure 9). Thus, the three mutants likely contain truncated QKY variants that are not properly located to the membrane. Membrane localisation, however, seems essential for QKY function as all three mutants show identical phenotypes, regardless of the number of C_2_ domains still present in the predicted truncated proteins. In summary, the results suggest that QKY is a membrane-bound Ca^2+^-signaling factor.

## Discussion

### *DOQ*, *QKY*, and *ZET* Contribute to *SUB*-Dependent Organ Development

In this work we set out to genetically identify additional components of the *SUB*-dependent mechanism regulating ovule development and floral morphogenesis. To this end we identified second-site mutations that resulted in a *sub*-like phenotype, reasoning that such mutations should reside in genes that either act directly in the *SUB* signaling pathway, or, that *SUB* and these genes affect parallel pathways that converge on a common molecular or developmental target. A defined set of criteria was applied in the identification of candidate mutants. At the developmental level, candidates were chosen that showed a *sub*-like ovule phenotype and twisting of petals, carpels, siliques and the stem. In addition, they were analysed for two types of *sub*-like cellular defects: an increased frequency of periclinal cell divisions in the L1/L2 layers of stage 3 floral meristems and a defect in root hair patterning. Mutations in three genes, *DOQ*, *QKY*, and *ZET*, satisfied all criteria and were considered as *sub*-like mutants.

Although *slm* mutants were phenotypically very similar there were some noteworthy distinctions. For example, *doq-1* was generally characterised by a weak ovule phenotype and mild silique/stem twisting. At the same time, *doq-1* showed the highest frequency of periclinal cell divisions in the L1/L2 layers of stage 3 floral meristems. Furthermore, rosette leaves of *doq-1* plants had elongated petioles and narrow leaf blades, features not observed in other *slm* mutants. These findings indicate that *DOQ* is less important for ovule, silique and stem development compared to other *SLM* genes, but plays a more prominent role in the regulation of cell shape/division plane in the young floral meristem and leaf development. In contrast *ZET* does not seem to be involved in leaf development as *zet* mutants exhibited apparently normal leaves. This clearly distinguishes *ZET* from the other *SLM* genes, which all seem to be important for leaf development. Plants with a defect in *ZET* activity also showed more defects in perianth development when compared to other *slm* mutants. Cell separation and disintegration observed in stage 3 floral meristems of *zet* mutants indicated that *ZET* may function in cell adhesion and viability, roles that seem to be unique among *SLM* genes.

Since the phenotypes of *sub* and the other *slm* mutants overlap we assessed the genetic relationship among *SLM* genes by testing whether the expression of *SUB* is sensitive to *SLM* activity. This was apparently not the case, at least not in a relevant manner, since *SUB* expression was unaltered in the respective other *slm* mutants in our transcriptome analysis and showed only minor downregulation in *slm* flowers when assayed by qRT-PCR. In addition, a functional *35S::SUB* transgene was unable to rescue the phenotype of *doq*, *qky* and *zet* mutants, including the floral defects. To further investigate the genetic interactions between *SLM* genes we performed a comprehensive double mutant analysis. The pleiotropic and variable phenotypes of *slm* single and double mutants complicated this task. Although an exaggerated phenotype was often observed in double mutants it was sometimes difficult to decide whether this represented an additive or synergistic phenotype. In addition, the various effects were often tissue dependent and thus precluded conclusions at the whole-plant level. One exception was *qky-8 zet-2* where *zet-2* appears to be largely epistatic to *qky-8*. This result indicates that *ZET* either acts upstream of *QKY* in a genetic pathway, or that *ZET* generally acts prior to *QKY*. Focusing on ovule development simplified our analysis, since a stringent set of morphological criteria could be applied. In *sub-1 zet-2*, *sub-1 qky-8*, and *doq-1 zet-2* double mutants a mass of cells with integument-like structures often developed in place of true ovules. Furthermore, all double mutants, with the exception of *qky-8 zet-2*, often showed aberrant inner integuments, in contrast to *slm* single mutants that only displayed defects in the outer integument. Taken together, these synergistic effects indicate that the *SLM* genes contribute to similar aspects of ovule development and show that *QKY* and *ZET* function is still present in ovules of *sub* plants. In addition, the analysis supports the notion that *DOQ* may play a more peripheral role in ovule development in comparison to *SUB*, *QKY*, and *ZET*. The exact genetic relationship between *SLM* genes during this process remains to be elucidated.

The phenotypic analysis of *slm* single mutants suggested that *SLM* genes variably contribute to several common functions but it also showed that they have distinct roles, both of which depend on tissue context. To characterise the molecular mechanisms that are under control of *SLM* genes and to narrow down the overlap of *SLM* gene function, we used transcript profiling of mRNA isolated from two different tissues. Our Z-score based meta-analysis revealed 89 and 193 largely non-overlapping *SUB*-responsive genes in the apex and flower data sets, respectively (Table 4). That we consistently observed larger numbers of affected transcripts in the flower data set might reflect the relatively higher importance of *SLM* genes for later stages of flower development, but it could also be explained by differences in the nature of the samples. While the flower sample only contained tissue from two well-defined floral stages, the apex sample contained the shoot apical meristem as well as flowers from stage 1 to stage 9 and thus was much more diverse. Consequently, the sensitivity of the array to detect changes that are limited to only a few developmental stages was reduced. When comparing the number of misregulated transcripts across the various genotypes, we found that fewer genes were affected in *sub* mutants than in the other *slm* mutants. This observation suggests that *DOQ*, *QKY* and *ZET* play broader roles than *SUB*. The result might have been expected for *ZET*, as *zet* mutants show the most severely affected flowers of all *slm* mutants, while *sub*, *qky* and *doq* mutant flowers, however, are more alike and thus their morphology is less congruent with a more dramatic change at the molecular level. Meta-analysis revealed a consistent overlap in misregulated genes between *sub* and the other *slm* mutants within a given tissue and even allowed us to identify a set of core targets shared by all four *SLM* genes. Interestingly, these transcripts were consistently up- or downregulated in all genotypes, while the direction of the change was dependent on tissue context. These results demonstrate not only that *SLM* genes affect common processes with a high tissue specificity, but also that the molecular mechanisms employed by *SLM* genes in the different tissues are distinct. In addition, the identification of common targets that show consistent behaviour across all genotypes underlines the sensitivity and specificity of our meta-analysis.

### *QKY* Encodes a Putative Transmembrane Protein Involved in Ca^2+^-Mediated Signaling

Sequence analysis of *QKY* suggests that QKY is the first described plant representative of the previously described MCTP family [65] and that it might act as a membrane-bound protein involved in a process regulated by Ca^2+^ and phospholipids. While the function of animal MCTPs is unknown, human MCTP2 is a membrane protein located to intracellular vesicular structures [65]. Interestingly, the C_2_ domains of human MCTPs were found to bind Ca^2+^ with high affinity but lacked any phospholipid binding capacity [65]. Other membrane-bound proteins with multiple C_2_ domains are the synaptotagmins and ferlins [70],[72]. Synaptotagmins contain two C_2_ domains and an amino-terminal transmembrane domain while most ferlins carry between four and six C_2_ domains and a carboxy-terminal transmembrane domain. Members of these two protein families function during regulated exocytosis, a process in which specific vesicles are signaled to fuse with the plasma membrane. Processes that rely on regulated exocytosis include neurotransmitter release at synapses and plasma membrane repair, a basic cellular process that mends physical injuries inflicted upon the plasma membrane [73]–[75]. During the repair process lysosomes [76] or specialised vesicles, such as the enlargosomes [77],[78], fuse with the plasma membrane providing new membrane material and facilitating resealing.

The synaptotagmins Syts 1 and 2 are required for Ca^2+^-regulated synaptic vesicle exocytosis during neurotransmitter release into the synaptic cleft [79],[80],[70] while Syt VII promotes lysosomal exocytosis during plasma membrane repair in fibroblasts [76]. The *C. elegans* ferlin FER-1 is localized to the membranes of membranous organelles (MOs) and promotes the fusion of MOs with the plasma membrane during the development of crawling spermatozoa [81],[82]. Mutations in dysferlin result in progressive muscular dystrophies [83]–[85] and dysferlin appears to be required for Ca^2+^-dependent sarcolemma resealing during membrane repair in skeletal muscle fibres [84],[86].

Given the predicted domain topology of QKY one could speculate that *QKY*, and possibly other *SLM* genes, could participate in the control of vesicle trafficking. In principle, the predicted membrane localisation of QKY also allows for the possibility that the SUB and QKY proteins interact directly. A role of *QKY* in vesicle trafficking would help to explain the non-cell-autonomy of *SUB* function in inter-cell-layer signaling [28]. Thus, in one possible scenario SUB could directly or indirectly regulate QKY which in turn might affect vesicular transport of factors mediating the non-cell-autonomy of *SUB*-dependent signaling. Further work needs to address this exciting hypothesis.

### Many *SLM*-Responsive Genes Relate to Cell Wall Biology, Hormone, and Stress Responses

To obtain additional insights concerning the molecular processes influenced by *SLM* function we investigated the known and predicted functions of *SLM*-responsive genes. The core sets of direct or indirect *SLM* targets included many genes encoding proteins relating to cell wall biology and a notable enrichment of genes that are inducible by hormones, such as JA and SA, and presumed to play a role in the plant's response to various stresses. For example, a group of genes misregulated in *slm* mutants encode proteins with a known or predicted role in the biosynthesis and/or function of the cell wall, such as MIOX2/4, GLP6, and putative glycine-rich proteins, lectin family proteins, and chitinases. Defects in *SLM*-activity also resulted in a deregulation of the basal expression of JA-inducible genes with a presumed role in abiotic stress responses to cold or dehydration. The altered expression of genes responsible for camalexin and glucosinolate metabolism, secondary metabolites involved in the defense against pathogens, indicates that *SLM*-activity appears to play a role in biotic stress responses as well.

The notion that *SLM* activity somehow relates to stress is reinforced by the interesting finding that a major group of *SLM*-responsive genes in flowers relate to various aspects of salicylic acid (SA)-dependent signaling. SA is synthesized upon exposure of plants to abiotic stresses, such as ozone or UV-C light, and plays an important role in pathogen defense (for reviews see for example [87]–[91]). Three of the *SLM*-responsive genes are known to be required for the pathogen-induced production of SA: *ICS1*/*SID2*/*EDS16* encodes an isochorismate synthase that synthesizes SA [92],[93], *EDS5*/*SID1* encodes a MATE family transporter potentially involved in the transport of SA intermediates [94], and *PBS3*/*GDG1* encodes an adenylate-forming enzyme required for SA accumulation [95],[96]. Other *SLM*-dependent genes are involved in further aspects of SA signaling. *EDS1* encodes a protein with a lipase-like domain [97], contributes to rapid SA accumulation in response to many SA-inducing stimuli, and is part of a central regulatory node of SA signaling. *NPR1*/*NIM1* constitutes another, though *SLM*-independent, central regulator of SA signaling. At low SA levels NPR1 is present in the cytoplasm as a homo-multimeric complex. Increased SA levels result in the dissociation of NPR1 oligomers into monomers, which enter the nucleus where they interact with specific TGA transcription factors. These NPR1-TGA complexes regulate expression of defense genes, such as *PATHOGENESIS-RELATED* (*PR*) genes and WRKY-type transcription factor genes. Interestingly, we found *NIMIN1* and *GRX480*, both of which act in the NPR1 pathway, to be downregulated in *slm* mutants. NIMIN1 encodes an interactor of NPR1 that counteracts NPR1 activity [98]. *GRX480* codes for a glutaredoxin that interacts with TGA2 [99] and is possibly involved in cross-talk between SA and JA signaling. Another important class of regulators implied in plant immunity, the regulation of *PR* genes and SA-mediated signaling are WRKY transcription factors. Interestingly, we found five *WRKY* genes to be downregulated in *slm* mutants and three of them are known direct targets of NPR1: *WRKY38*, *WRKY53*, and *WRKY70* [100]. *WRKY53* is a positive modulator of systemic acquired resistance (SAR) [100] while *WRKY70* is involved in the cross-talk between SA and JA responses by acting as a negative regulator of JA-inducible genes [101],[102]. In addition, *WRKY70* is required for resistance against several bacteria, fungi and an oomycete [100],[101],[103],[104]. Another *SLM*-responsive *WRKY* gene, *WRKY33*, is a positive regulator of resistance against the necrotrophic fungi *Botrytis cinerea* and *Alternaria brassicicola* [105]. Fitting in the overall picture was the observation that expression of five *PR* genes was reduced in *slm* mutants, including *PR1*, *PR2*, and *PR5* whose activities often undergo coordinated changes in response to various stimuli including SA [106]. In addition, *PR1* is likely a direct target of a NPR1/TGA complex [107],[108].

Many of the *SLM*-responsive genes involved in SA-signaling and pathogen defense are normally transcribed at basal levels while their expression is induced several fold in the case of pathogen attack and increased levels of SA. Our transcriptome analysis of *slm* mutants suggests that *SLM* function is required for basal steady-state expression of SA-responsive genes. Thus, *SLM* function could be involved in priming components of the cellular defense machinery, thereby enabling floral cells to respond faster to invading pathogens. In this scenario *SLM* genes would contribute to basal resistance.

Another scenario is based on the hypothesis that *QKY* may play a role in membrane traffic. Since membrane repair or cell wall synthesis require extensive vesicle trafficking [74],[109] *SLM*-dependent processes could be involved in the regulation of the composition of the plasma membrane or the cell wall and alterations in the two cellular compartments of *slm* mutants could be interpreted as wounding stress. For example, it was suggested that the *SLM*- and SA-responsive *WALL-ASSOCIATED RECEPTOR KINASE1* (*WAK1*) gene transmits changes in the cell wall to the interior of the cell. In addition, *WAK1* protects plant cells against negative aspects of the pathogen response [110]. The RLK THESEUS1 (THE1) has been proposed to act as a sensor of cell wall integrity [111]. It was shown that in the absence of proper cellulose synthesis *THE1* modulates the activity of genes affecting pathogen defense, cell wall cross-linking and stress responses. Although *THE1* and the *SLM* genes do not share common transcriptional targets, they affect a functionally related spectrum of genes. Thus, it is conceivable that one aspect of *SLM* function relates to the surveillance of cell wall integrity or the repair of plasma membranes.

The two proposed models are not mutually exclusive and it will be an exciting challenge to further dissect the biological function of *SLM* genes in development, cell biology, and stress response.

## Materials and Methods

### Plant Work and Genetics

*Arabidopsis thaliana* (L.) Heynh. var. Columbia (Col-0) and var. Landsberg (*erecta* mutant) (L*er*) were used as wild-type strains. The *sub-1* mutant was described previously [25]. Plants were grown in a greenhouse under Philips SON-T Plus 400 Watt fluorescent bulbs on a long day cycle (16 hrs light). Dry seeds were sown on soil (Patzer Einheitserde, extra-gesiebt, Typ T, Patzer GmbH & Co. KG, Sinntal-Jossa, Germany) overlying perlite, stratified for 4 days at 4°C and then placed in the greenhouse. Plant trays were covered for 7–8 days to increase humidity and support equal germination. L*er* seeds mutagenized with ethylmethane sulfonate (EMS) were obtained from Lehle Seeds (Round Rock, TX, USA). 60'000 M2 plants, corresponding to about 7'500 M1 plants, were screened for plants exhibiting a *sub*-like phenotype. All *sub*-like mutants described in this paper were outcrossed three times to L*er* prior to further analysis. Two *qky* T-DNA insertion mutants (line SALK_140123 and SALK_043901) [112] were obtained from the ABRC (http://www.arabidopsis.org). The *GL2::GUS* line [33] in L*er* was crossed into *slm* mutants for analysis of root hair specification.

### Recombinant DNA Work

For DNA and RNA work standard molecular biology techniques were used [113]. PCR-fragments used for cloning were obtained using either PfuUltra high-fidelity DNA polymerase (Stratagene) or TaKaRa PrimeSTAR HS DNA polymerase (Lonza, Basel, Switzerland). All PCR-based constructs were sequenced. Information regarding all primers used in this study is given in supporting Table S1.

### Generation of the *35S::SUB* Construct

In order to generate a translational fusion between SUB and the small c-myc tag *SUB* cDNA was amplified by PCR from plasmid H2H6T7 [25] using primers SUB-CmycF and SUB-Cmyc-R. A 3xmyc-tag was amplified from a pFastBac-HT A plasmid (Invitrogen) containing a 2xmyc tag using primers cmyc-F and cmyc-R. Both PCR fragments were gel purified and an overlap PCR was set up using primers SUB-Cmyc-F and cmyc-R. The overlap PCR product was cloned into vector PCRII TOPO (Invitrogen) and was designated PCRII TOPO SUB:3xmyc. SUB:3xmyc was then cloned into pCAMBIA2300 (www.cambia.org). To this end pCAMBIA2300 was digested with *Bam*HI and *Pml*I to remove the 35S::GUS cassette. PCRII TOPO SUB:3xmyc was digested with *BamH*I and *Bsr*BI and the resulting SUB:3xmyc fragment was cloned into *Bam*HI/*Pml*I-digested pCAMBIA2300 resulting in plasmid SUB:3xmyc pCAMBIA2300. To obtain the 35S promoter [114] the vector pART-7 [115] was digested with *Not*I, blunt-ended by T4 DNA polymerase treatment and the 35S:NOS cassette was isolated by gel purification and digested with *Xba*I, resulting in a 35S promoter fragment with a 5′ blunt and 3′ sticky end. SUB:3xmyc pCAMBIA2300 was blunt-ended after *Spe*I digestion at the 5′ end and redigested with *Bln*I to create an *Xba*I-compatible 3′ end. Ligation resulted in the final *35S::SUB:myc* construct (abbreviated *35S::SUB*). The plasmid was verified by sequencing.

### Generation of *35S::SUB slm* Plants

Transformation of *sub-1*, *doq-1*, *qky-8* and *zet-2* mutants with the *35S::SUB* construct was performed using the floral dip method [116] and *Agrobacterium tumefaciens* strain GV3101 [117]. Transgenic T1 plants were selected on 50 µg/ml Kanamycin plates and subsequently transferred to soil for phenotypic inspection. About 41 percent of the independent *35S::SUB sub-1* T1 plants scored showed a wild-type phenotype (51/126 scored). This indicates that the c-myc tag at the carboxy-terminus of SUB may result in a reduction of SUB functionality. To assay transgene expression RNA was isolated from inflorescences using the NucleoSpin RNA II kit (Macherey-Nagel, Düren, Germany). First-strand cDNA was synthesized from 2 µg of total RNA using Moloney Murine Leukemia Virus (M-MuLV) reverse transcriptase (New England Biolabs, Frankfurt, Germany). Semi-quantitative PCR was performed using *Taq* DNA polymerase (New England Biolabs, Frankfurt, Germany) and a transgene-specific primer pair (sRT-SUBcmyc_F, sRT-SUBcmyc_R). Between 24 and 32 thermal cycles were tested. The *GAPC* gene was used as positive control [118].

### Map-Based Cloning of *QKY*

To map the *QKY* locus at high resolution, an F2-mapping population was generated. F1 plants from *qky/qky* (L*er*) and *QKY/QKY* (Col) crosses were allowed to self-pollinate, and the F2 progeny were screened for *qky* individuals based on twisted inflorescence morphology. DNA was isolated and used for PCR-based amplification of molecular markers. Indel polymorphism data was derived from the Monsanto L*er* sequence database at TAIR [119]. Primer sequences for indel and CAPS markers are shown in supporting Table S1. Marker amplifications from 598 mutant individuals restricted the *qky* map interval to 103 kb, as defined by markers F25A4(*Bgl*II) and 27.99(*Rsa*I). Candidate genes were analysed via T-DNA insertion mutant analysis and/or sequence determination revealing that At1g74720 carried mutations in various *qky* alleles. To confirm *qky* allelic mutations, nucleotide sequences were obtained from both strands of PCR-amplified fragments. In all three EMS-induced *qky* alleles transitions result in stop codons. The *qky-7* allele carries a G to A transition at position 2229 (genomic DNA, relative to the start ATG triplet (+1)) resulting in a shorter predicted protein (W743*). The *qky-8* allele carries another G to A transition at position 2706 (W902*), and in *qky-9* a C to T transition was found at position 649 (Q217*). We also determined the genomic integration sites of two T-DNA insertions in At1g74720: SALK_140123 is located at position 2576 and SALK_043901 at position 3056. Both lines exhibit a *qky* phenotype. SALK_140123 is predicted to carry a shorter QKY protein of 878 residues with the 29 last residues (RSHKGSHVMTPADDAGQAVLRLELTEPQR*) being encoded by the T-DNA. SALK_043901 results in a predicted shorter protein of 1022 residues with residues 1019–1022 (LFVV*) being encoded by the T-DNA.

Near full-length *QKY* cDNA sequence was assembled from sequences derived from four publicly available RIKEN RAFL cDNA clones [120]. The cDNA clones partially overlapped (RAFL16-35-G10, RAFL22-02-B15, RAFL22-66-H20, RAFL22-96-F19) with one clone (RAFL22-96-F19) containing the 3′ poly(A) stretch. Additional 5′ RACE experiments [121] did not result in more extended 5′ cDNA sequences and comparisons of the available *QKY* genomic and cDNA sequences did not reveal introns.

### Bioinformatic Analysis

Protein domain searches were conducted using the PFAM database [64]. Transmembrane topology was predicted using the TMHMM webserver [122].

### Quantitative Real-Time PCR

Tissue was harvested as described for the whole-genome transcriptome analysis. RNA was extracted as outlined above. First-strand cDNA was synthesized from 1.5 µg of total RNA via reverse transcription, using the Transcriptor High Fidelity cDNA Synthesis Kit (Roche Diagnostics GmbH, Mannheim, Germany). Quantitative real-time PCR was performed on a Roche LightCycler using the SYBR Green I detection kit according to the manufacturer's recommendations (Roche). Amplification of *UBC21*/At5g25760 served as a normalization control [123]. Primer sequences are summarized in supporting Table S1. Using the comparative Ct method, all gene expression levels were calculated relative to *UBC21*.

### Microscopy and Art Work

Preparation and analysis of propidium iodide-stained samples for confocal laser scanning microscopy, scanning electron microscopy, and histochemical localisation of ß-glucuronidase (GUS) activity in whole-mount tissue was done essentially as described [124], [33], [125]–[128]. Confocal laser scanning microscopy was performed with an Olympus FV1000 setup using an inverted IX81 stand and FluoView software (FV10-ASW version 01.04.00.09) (Olympus Europa GmbH, Hamburg, Germany). After excitation at 488 nm with a multi-line argon laser, propidium iodide fluorescence (580–630 nm slit width) and autofluorescence (500–530 nm slit width) was detected. One-way scan images (scan rate 12.5 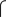
s/pixel, 512×512 pixels, Kahlman frame, average of four scans) were obtained using an Olympus 40× objective (UApo/340 40×/1.35 Oil Iris). Confocal Z-stack imaging of floral meristems was performed using 1.5 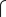
m sections. Plants or various plant organs were analysed under an Olympus SZX12 stereo microscope. Images were obtained using a ColorView III digital camera (Olympus) and Cell∧P software (version 2.4 build 1131, Olympus), saved as TIFF files, and adjusted for color and contrast using Adobe Photoshop CS3 (Adobe, San Jose, CA, USA) software.

### Whole-Genome Transcriptome Analysis and Global Expression Profiling

Tissue was harvested from 21-day plants grown in continuous light at 23°C. Wild type and mutants were in the L*er* background. The inflorescence apex plus flowers up to stage 9 were kept separate from later stage 10–12 flowers (the oldest 3–4 closed flower buds). The tissue was immediately frozen in liquid nitrogen and stored at −80°C. RNA was extracted with the Plant RNeasy Mini kit (Qiagen). 1 µg of total RNA was used for probe synthesis with the MessageAmp II-Biotin Enhanced kit (Ambion) according to the manufacturer's instructions. Affymetrix ATH1 GeneChips were hybridised, washed, and stained as described [129]. The gcRMA implementation in the Bioconductor and R software packages was used for background correction, quantile normalization and expression estimate computation [130],[131] (www.bioconductor.org) [132]. Expression estimates were transformed to linear scale and the fold change (FC) was calculated for each gene in each mutant-wildtype comparison by dividing the expression estimate of the mutant condition by the value of an according control. Comparisons were done on a single replicate basis for all possible pair wise comparisons. A Z-score for each fold change was calculated by dividing the difference of log_2_ transformed FC and the mean of the FC population by the standard deviation of the FC population (
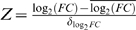
). Genes were considered as differentially expressed if they displayed a Z-score>1 in a single comparison and an average Z-score>2 over all comparisons or, if they displayed a Z-score<−1 in a single comparison and an average Z-score over all comparisons <−2.

### Accession Numbers

The *QKY* cDNA sequence was deposited at GenBank under the accession number FJ209045. The expression profile data have been deposited to the EMBL-EBI ArrayExpress repository under the accession E-MEXP-1592.

## Supporting Information

Dataset S1 This list details the 14 genes that show significant changes in expression level in all L*er* versus *sub-like mutant (slm)* comparisons. This list thus comprises the apex core target gene list.
(0.03 MB XLS)

Dataset S2 This list details the 93 genes that show significant changes in expression level in all L*er* versus *sub-like mutant (slm)* comparisons. This list thus comprises the flower core target gene list.
(0.07 MB XLS)

Table S1 List of primers
(0.08 MB DOC)
