# Supplementary material for: DETORQUEO, QUIRKY, and ZERZAUST Represent Novel Components Involved in Organ Development Mediated by the Receptor-Like Kinase STRUBBELIG in Arabidopsis thaliana
Source: PLoS Genet. 2009 Jan 30;5(1):e1000355. doi: 10.1371/journal.pgen.1000355 (PMC2628281; doi:10.1371/journal.pgen.1000355)
Supplement: Table S1 — List of primers (0.08 MB DOC) [file pgen.1000355.s003.doc]

Table S1. Primer List

| Primer Name | Primer (F and R) | Product Sizes |
| --- | --- | --- |
| Mapping markers |  |  |
| F10O3(48ID)_F ^1^  F10O3(48ID)_R ^1^ | 5’-TTTCCCAGTTTCATTTAGACATTTC-3’  5’-GCTAATTTGTTAAGAAGGATTATGG-3’ | Ler (597 bp)  Col (645 bp) |
| CER453151(58ID)_F ^1^  CER453151(58ID)_R ^1^ | 5’-GCTCTGTTAGGTACGCCTTTTGTTACAAAC-3’  5’-GTGAGTAACGTGCATGTTGTTGGAATC-3’ | Ler (171 bp)  Col (229 bp) |
| F13011(164ID)_F^1^  F13011(164ID)_R^1^ | 5’-AGTGATTGGATGGTCGGTATG-3’  5’-TGGTTTTGGTGAGTTCTGCT-3’ | Ler (330 bp)  Col (494 bp) |
| F25A4(*Bgl*II)_F ^2^  F25A4(*Bgl*II)_R ^2^ | 5’-GTTTGGTGGCTCCGTTGTCATAAT-3’  5’-TGGCAAAGCAGGAGAGGAAACAT-3’ | Ler (266, 192 bp)  Col (458 bp) |
| 27.99(*Rsa*I)_F ^2^  27.99(*Rsa*I)_R ^2^ | 5'-AATGGTCTTATTGTTCCGGTTT-3', -  5'-CAGAACCATGATGCTTTCCA-3' | Ler (457 bp)  Col (356 bp, 101 bp) |
|  |  |  |
| RT-PCR |  |  |
| sRT-SUBcmyc _F  sRT-SUBcmyc _R | 5’-TCACCAGAATTTCAAGTCTTCCAA-3’  5’-CCGACGTCAGGGCCCCGATC-3’ | 456 bp |
| GAPC _F  GAPC _R | 5’-CACTTGAAGGGTGGTGCCAAG-3’  5’-CCTGTTGTCGCCAACGAAGTC-3’ | 543 bp |
|  |  |  |
| Real time PCR |  |  |
| RT_SUB_R  RT_SUB_L | 5’-TGGCATTGGATACCCATATTT-3’  5’-ACCCCCACTATGGGAAAAGT-3’ | 93 bp |
| RT3-QKY_F  RT3-QKY_R | 5’-GAAGGCGAAAAACGGAGGG-3’  5’-GAGGCGTCAGAGAACATCC-3’ | 110 bp |
| RT_25760_R  RT_25760_L | 5’-GCGAGGCGTGTATACATTTG-3’  5’-TCCTCTTAACTGCGACTCAGG-3’ | 87 bp |
|  |  |  |
| *QKY* genomic rescue |  |  |
| *Kpn*I/QKYp_F | 5'-CGGGGTACCAGAAAGAGTAACATTTGTATTTGATTGT -3’ | NA |
| QKY/*Xba*1_R | 5'-GCTCTAGATGAAGAAAAATCGAAGCGAAGCGATGA-3’ | NA |
|  |  |  |
| 35S::SUB transgene |  |  |
| cmyc-F | 5’-GGGGCCCTGACGTCGGTCTCGAGAATGGAGCAAAAGCTCA-3’ | NA |
| cmyc-R | 5’-TTATTCATTCAAGTCCTCTTCAGAAATGAGCTTTTGCTC C-3’ | NA |
| SUB-CmycF | 5’-CCTAGG AGG GCGGCCATGAGCTTTACAAGATGGGAAGTGTTC-3’ | NA |
| SUB-CmycF | 5’-GAGACCGACGTCAGGGCCCCGATCATATGTTGAAGATCTTGG-3’ | NA |
|  |  |  |

^1^ Indel-based molecular marker, ^2^ RFLP-based molecular marker. NA, not applicable
